# Capturing Differential Allele-Level Expression and Genotypes of All Classical HLA Loci and Haplotypes by a New Capture RNA-Seq Method

**DOI:** 10.3389/fimmu.2020.00941

**Published:** 2020-05-29

**Authors:** Fumiko Yamamoto, Shingo Suzuki, Akiko Mizutani, Atsuko Shigenari, Sayaka Ito, Yoshie Kametani, Shunichi Kato, Marcelo Fernandez-Viña, Makoto Murata, Satoko Morishima, Yasuo Morishima, Masafumi Tanaka, Jerzy K. Kulski, Seiamak Bahram, Takashi Shiina

**Affiliations:** ^1^Department of Pathology, Stanford University School of Medicine, Palo Alto, CA, United States; ^2^Department of Molecular Life Science, Tokai University School of Medicine, Isehara, Japan; ^3^Faculty of Health and Medical Science, Teikyo Heisei University, Toshima-ku, Tokyo, Japan; ^4^Division of Hematopoietic Cell Transplantation, Department of Innovative Medical Science, Tokai University School of Medicine, Isehara, Japan; ^5^Histocompatibility, Immunogenetics, and Disease Profiling Laboratory, Stanford Blood Center, Stanford Health Care, Palo Alto, CA, United States; ^6^Department of Hematology and Oncology, Nagoya University Graduate School of Medicine, Nagoya, Japan; ^7^Division of Endocrinology, Diabetes and Metabolism, Hematology, Rheumatology, Second Department of Internal Medicine, Graduate School of Medicine, University of the Ryukyus, Nishihara, Japan; ^8^Department of Promotion for Blood and Marrow Transplantation, Aichi Medical University School of Medicine, Nagakute, Japan; ^9^Faculty of Health and Medical Sciences, The University of Western Australia Medical School, Crawley, WA, Australia; ^10^Laboratoire d'ImmunoRhumatologie Moléculaire, Plateforme GENOMAX, INSERM UMR_S 1109, LabEx TRANSPLANTEX, Fédération Hospitalo-Universitaire OMICARE, Laboratoire International Associé INSERM FJ-HLA-Japan, Fédération de Médecine Translationnelle de Strasbourg (FMTS), Faculté de Médecine, Université de Strasbourg, Service d'Immunologie Biologique, Nouvel Hôpital Civil, Strasbourg, France

**Keywords:** human leukocyte antigen, next-generation sequencing, HLA allele, RNA expression level, genotyping, capture RNA-Seq

## Abstract

The highly polymorphic human major histocompatibility complex (MHC) also known as the human leukocyte antigen (HLA) encodes class I and II genes that are the cornerstone of the adaptive immune system. Their unique diversity (>25,000 alleles) might affect the outcome of any transplant, infection, and susceptibility to autoimmune diseases. The recent rapid development of new next-generation sequencing (NGS) methods provides the opportunity to study the influence/correlation of this high level of HLA diversity on allele expression levels in health and disease. Here, we describe the NGS capture RNA-Seq method that we developed for genotyping all 12 classical HLA loci (*HLA-A, HLA-B, HLA-C, HLA-DPA1, HLA-DPB1, HLA-DQA1, HLA-DQB1, HLA-DRA, HLA-DRB1, HLA-DRB3, HLA-DRB4*, and *HLA-DRB5*) and assessing their allelic imbalance by quantifying their allele RNA levels. This is a target enrichment method where total RNA is converted to a sequencing-ready complementary DNA (cDNA) library and hybridized to a complex pool of RNA-specific HLA biotinylated oligonucleotide capture probes, prior to NGS. This method was applied to 161 peripheral blood mononuclear cells and 48 umbilical cord blood cells of healthy donors. The differential allelic expression of 10 HLA loci (except for *HLA-DRA* and *HLA-DPA1*) showed strong significant differences (*P* < 2.1 × 10^−15^). The results were corroborated by independent methods. This newly developed NGS method could be applied to a wide range of biological and medical questions including graft rejections and HLA-related diseases.

## Introduction

The highly polymorphic human major histocompatibility complex (MHC), also known as the human leukocyte antigen (HLA), expresses class I and II molecules (alleles) that present antigens to the T-cell receptors as part of the adaptive immune response (1–4). The high level of gene sequence diversity [25,756 alleles and counting; IPD IMGT/HLA database (Release 3.38.0), http://hla.alleles.org/nomenclature/stats.html] within the HLA system may govern the outcome of many transplants (tolerance or rejection) (5, 6), infections, susceptibility to autoimmune diseases (2, 7–9), and allergic reactions to various drugs (10). Moreover, the efficacy of recently developed checkpoint inhibitory therapies in immuno-oncology appear to be directly linked to the so-called “tumor mutation burden” that is the status of neo-antigens presented by the patient's HLA class I alleles (4). During the past 20 years, although HLA allele studies have shifted from serological allele typing to molecular genotyping, most have still focused on the phenotypic description of association between diseases and HLA alleles (11, 12).

With the continuing next generation sequencing (NGS) revolution, a better understanding is slowly emerging about the diversity of the HLA genomic and transcriptomic regions including the qualitative and quantitative effects of regulatory variation on HLA expression, gene diversity, and polymorphisms (alleles) on shaping lineage-specific expression, and HLA expression on disease susceptibility and transplantation outcomes. Regulatory *cis* and *trans* polymorphisms that affect transcriptional regulation and susceptibility to complex diseases (13) are considered to be a driving force in phenotypic evolution (14, 15). Previous small-scale, low-resolution, targeted studies revealed the importance of differential allelic expression (DAE) of HLA genes in disease development and progression. Cauli et al. (16) reported a greater expression of HLA-B27 molecules in patients with ankylosing spondylitis than in healthy subjects. The association among allelic differences in HLA expression levels and disease were reported for single HLA alleles/loci such as HLA-B expression and immunoglobulin A (IgA) deficiency (17); HLA-C expression and HIV control (18–20); Crohn disease (21), and acute graft-vs.-host disease (GVHD) (22); HLA-DQ and HLA-DR expression and cystic fibrosis (23); HLA-DP expression and hepatitis B virus infection (24) and acute GVHD (25); and HLA-DRB5 and interstitial lung disease (26). In addition, suppressed or abnormal HLA expression levels were reported in gastric cancer (27), cancer cell lines (28), ovarian carcinomas (29), Merkel cell carcinoma (30), and lung cancer (31). Although polymorphisms located in the 5′ promoter region and 3′ untranslated regions (3′UTR) of HLA genes can affect HLA expression levels (21, 32–36), reliable data on HLA polymorphisms associated with HLA gene expression levels in HLA-associated disease, infection, and transplantation are still lacking.

There are different ways to measure HLA differential allele expression in leukocytes. Previously, a few particular HLA genes and alleles were examined in expression studies using flow cytometry and fluorolabeled monoclonal antibodies to measure the intensity of HLA protein surface expression (20, 21, 37) and by quantitative reverse transcription PCR (qRT-PCR) to estimate HLA transcription levels (38). Microarray methods, such as Affymetrix and Illumina, using oligoprobes are useful for the semiquantification of HLA gene transcripts expressed by a larger array of HLA class I and II genes (39, 40), but like flow cytometry and qRT-PCR, they do not identify the different HLA genotypes and alleles. In addition, all these methods are labor intensive/time consuming and often lead to ambiguous results because of problems with specificity and sensitivity and inadequate controls and reference samples. New RNA quantitative techniques based on RNA-sequencing (RNA-Seq) have emerged recently (41), and genotyping, mapping the expression quantitative trait locus, and analyzing allele-specific expression from public RNA-Seq data are promising new development (42). In addition, a computational pipeline to accurately estimate expression for HLA genes based on RNA-Seq was developed for both locus-level and allele-level estimates (43).

HLA genes also can be genotyped by amplicon sequencing using HLA transcripts as reverse-transcribed complementary DNA (cDNA) (44) and HLA RNA expression levels quantitated by amplicon sequencing using HLA locus-specific primers (45). However, the method using HLA locus-specific primers for measuring RNA levels are mostly semiquantitative because PCR efficiency can differ between the polymorphic HLA alleles (46). In contrast, a recently described capture RNA-Seq method for the quantitation of RNA expression levels of targeted genes was shown to provide enhanced coverage for sensitive gene discovery, robust transcript assembly, and accurate gene quantification (47).

In the present paper, we describe a newly developed capture RNA-Seq method for enriched NGS, genotyping, and for quantitating RNA levels of all 12 classical HLA loci [*HLA-A, HLA-B, HLA-C, HLA-DPA1, HLA-DPB1, HLA-DQA1, HLA-DQB1, HLA-DRA, HLA-DRB1, HLA-DRB3, HLA-DRB4*, and *HLA-DRB5* (*HLA-DRB3/DRB4/DRB5*)] and alleles using over 200 RNA samples isolated from the peripheral blood mononuclear cells (PBMCs, *n* = 161) and umbilical cord bloods (UCBs, *n* = 48) of healthy donors.

## Materials and Methods

### Sample Information

A reference set of PBMC samples from 161 donors were selected from a larger number of high-resolution genotyped samples obtained from 2,344 donors recruited for bone marrow transplantation (BMT) as part of the Japan Marrow Donor Program (JMDP) (48). This sample of genotyped Japanese donors represented more than 99.2% cumulative allele frequency (138 alleles) of the known HLA alleles in Japanese population at the field-2 level of resolution (an allele resolution level of sequences that differs by a non-synonymous substitution) at six HLA loci (20 alleles for *HLA-A*, 37 *HLA-B*, 19 *HLA-C*, 30 *HLA-DRB1*, 16 *HLA-DQB1*, and 16 *HLA-DPB1*) (Table S1). A total of 48 UCB samples previously registered and stored at the Tokai University Cord Blood Bank also were used in this study. The high-resolution HLA genotyping data for these UCB was unknown before this study. The mononuclear cells of the UCB were isolated by Ficoll–Paque density separation (Ficoll–Paque™ Plus, GE Healthcare).

### Isolation of RNA Samples and Measurement

Total RNA was isolated from the PBMC and UCB mononuclear cells using TRIzol (Thermo Fisher Scientific). The quantity and quality of the RNA were determined using an RNA 6000 Nano Kit with a Bioanalyzer 2100 (Agilent Technologies).

### Design of Sequence Capture Probes

The customized biotinylated nucleotide capture probes were designed and synthesized by Roche's proprietary method for use with the SeqCap RNA Enrichment System (Roche, NimbleGen, KAPA Biosystems). The exact number of 5′-biotinylated probes used per gene locus for this study was not released to us by the manufacturer. However, each of the single-stranded, 5′-biotinylated capture oligonucleotide probes in the synthesized set was 50–100 bases (average 75 bases) in length. Taken together, all of the capture probes in the set represented sequences of 172 alleles (19 *HLA-A*, 39 *HLA-B*, 19, *HLA-C*, 2 *HLA-DRA*, 31 *HLA-DRB1*, 3 *HLA-DRB3*, 3 *HLA-DRB4*, 3 *HLA-DRB5*, 16 *HLA-DQA1*, 15 *HLA-DQB1*, 4 *HLA-DPA1*, and 18 *HLA-DPB1*) that were representative of the Japanese population (Table S2) (49). Of the 172 allelic targets, 160 covered full-length HLA regions and 12 sequences covered partial regions such as specific exons and/or intron regions, respectively. The total nucleotide length covered by the designed probes was 1,321,811 bp, which covered 96.1% (1,270,384 bp) of the targeted regions. The remaining 3.9% of the targeted regions were omitted from the probe design and synthesis because of repeat sequences.

### Sequence Capture and Next-Generation Sequencing

The workflow for the Capture RNA-Seq profiling by NGS is shown in Figure S1. The basic steps were (1) RNA fragmentation, (2) preparation of reverse-transcribed RNA libraries, (3) hybridization with biotin-labeled capture probes containing target sequences, (4) capture and enrichment of targeted sequences with streptavidin-coated paramagnetic beads, (5) library amplification, (6) NGS Illumina sequencing, and (7) data analysis for HLA allele assignments and quantitation of allelic sequence expression (Figure S1A).

Total RNA (100 ng) was fragmented by shearing and reverse transcribing into cDNAs by second-strand synthesis using a KAPA Stranded RNA-Seq Library Preparation Kit. The sheared product was purified by Agencourt AMPure XP reagent (Beckman Coulter), and the cDNA libraries were constructed using KAPA library preparation kits (KAPA Biosystems) with SeqCap Adapter Kits A and B (Roche Life Science). The cDNA libraries were sized and quantitated using an Agilent DNA 1000 Kit with the Bioanalyzer 2100 (Agilent Technologies). One hundred nanograms of each indexed library was pooled together according to the manufacturer's recommendation. The pooled library was mixed with a SeqCap HE universal oligonucleotide, SeqCap HE-Oligo Kits A and B (Roche Life Science), and COT-1 human DNA and then vacuum evaporated at 60°C for ~30 min. The custom-designed sequence capture probe set was added and hybridized at 47°C for 18 h using a SeqCap Hybridization solution in a GeneAmp PCR System 9700 (Thermo Fisher Scientific). After hybridization, the non-specific hybridization products were washed out with a Wash Kit, and the captured library was enriched with a SeqCap Pure Capture Bead Kit (Roche/NimbleGen). The enriched beads were subjected directly to post-capture amplification by ligation mediated (LM) PCR using a SeqCap EZ accessory kit v2 (Roche/NimbleGen). The enriched and amplified NGS library was purified by AMPure XP reagent, quantitated using the Agilent DNA 1000 Kit with the Bioanalyzer 2100, and sequenced using MiSeq Reagent Kit v.2 (300 cycles), generating 150 bp pair-end sequence reads with an Illumina MiSeq System according to the manufacturer's protocol (Illumina).

### Data Processing and Allele Assignment of HLA Genotypes

After the sequencing runs, basic sequence information such as the read numbers and quality values were calculated with a FASTX_quality_stats program included in a FASTX-Toolkit package (ver. 0.0.13) for short-read data preprocessing (http://hannonlab.cshl.edu/fastx_toolkit/).

The FASTQ sequence files for each sample were used for HLA genotyping and allele assignment up to the field-3 level (an allele resolution where synonymous and/or non-synonymous DNA substitutions in the coding region define alleles). The HLA alleles for the 12 classical HLA loci, *HLA-A, HLA-B, HLA-C, HLA-DRA, HLA-DRB1, HLA-DRB/DRB4*/*DRB5, HLA-DQB1, HLA-DQA1, HLA-DPA1*, and *HLA-DPB1*, were assigned by nucleotide similarity searches in the IPD-IMGT-HLA database (http://hla.alleles.org/) using the BLAT program (50), included in an in-house Sequence Alignment Based Assigning Software (SeaBass) (51). When novel single-nucleotide polymorphisms (SNPs) were detected, they were confirmed by Sanger direct sequencing using newly designed sequencing primers.

### Calculation and Normalization of Sequence Read Numbers (RNA Levels)

Mapping of the reads and the HLA allele sequences assigned by the BLAT search as references were performed using GS Reference Mapper Ver. 3.0 software (Roche). To precisely extract in-phase read numbers that are our measure of RNA levels, we limited the mapping regions to highly polymorphic exons: 546 bp of exons 2 and 3 in *HLA-A, HLA-B*, and *HLA-C*; 239 bp of exon 2 in *HLA-DRA*; 270 bp of exon 2 in *HLA-DRB1, HLA-DRB3, HLA-DRB4, HLA-DRB5*, and *HLA-DQB1*; 249 bp of exon 2 in *HLA-DQA1*; 246 bp of exon 2 in *HLA-DPA1*; and 264 bp of exon 2 in *HLA-DPB1*. The mapping parameter was set to a 100% matched condition between the reads and the references to avoid mismapping among the HLA loci and contamination of *in vitro* generated PCR crossover products (51, 52).

In order to compare differences of the RNA levels (cDNA sequence read numbers) among the HLA loci and alleles, we normalized the mapped read numbers as follows. The mapped raw read numbers of each allele at each locus were first standardized by their target sizes. To derive the “normalized read numbers” within a particular set of loci, the total size-standardized read numbers included in the set were estimated, and the total read numbers were standardized to 1 million. Then, the normalized read numbers of each allele at each locus in the set were calculated as relative read numbers in 1 million total reads. An example of deriving the normalized read numbers at the *HLA-A, HLA-B*, and *HLA-C* loci is described in Figure S1B.

For comparison of the RNA levels expressed by the same or different HLA genes and alleles, we prepared the following four datasets: (1) normalized read numbers of the 12 HLA loci and alleles (including two *HLA-DRB3/DRB4/DRB5* alleles) using 78 PBMC and 18 UCB samples, (2) normalized read numbers of the HLA class I loci and alleles using 161 PBMC and 48 UCB samples, (3) normalized read numbers of the HLA class II loci (excluding *HLA-DRB3/DRB4/DRB5*) and alleles using 161 PBMC and 48 UCB samples, and (4) normalized read numbers of the HLA class II loci and alleles (including two *HLA-DRB3/DRB4/DRB5* alleles) using 78 PBMC and 18 UCB samples (Figure S1C).

Comparative analyses of the read numbers (RNA levels) among the HLA loci and among the HLA alleles were carried out using only heterozygous alleles that were completely phased in each dataset (Figure S1C). We excluded homozygous alleles, partially phased alleles, and hemizygous alleles from this study to avoid mapping biases because it was not possible to divide these reads into unphased exonic regions.

### Statistical Analyses

The results of the comparative analyses of capture RNA sequences were drawn as a box-and-whisker diagram using the graphics output of Microsoft Excel 2016. The box-and-whisker diagram displays the median with upper and lower quartiles within the box, with the whiskers extended 1.5 times the interquartile range from the box. Statistical differences of the RNA levels expressed by the HLA loci and alleles were calculated by analysis of variance (ANOVA) in Microsoft Excel 2016. A two-sided *p* < 0.05 was considered statistically significant. Bias in statistical significance due to multiple calculations was not taken into account. Correlation coefficients, coefficient of determination, and approximate curve between different sets of results were calculated by the Pearson correlation coefficient method using the Microsoft Excel function (Excel for Mac version 16.25).

## Results

### Assay Design

We devised a capture-based RNA-Seq NGS method to simultaneously analyze DNA alleles and RNA expression levels of HLA genes as described in section Material and Methods. Because target sequences have a high degree of polymorphisms, we designed a capture probe set, which covers 172 most frequent Japanese HLA allele sequences. The NGS data that we obtained were processed by an in-house program, in which only the reads showing perfect matches (100% identity) to reference sequences were included in the analyses. The relative levels of RNA expression of each HLA gene were deduced from read numbers, which were normalized against total read numbers of a set of HLA genes, among which RNA expression levels were compared (see section Material and Methods). Thus, we obtained four data sets: set 1, composed of all 12 HLA loci; set 2, composed of *HLA-A, HLA-B*, and *HLA-C* loci; set 3, composed of class II loci excluding *HLA-DRB3/DRB4/DRB5* genes; set 4, composed of class II loci including *HLA-DRB3/DRB4/DRB5* genes (see section Material and Methods; Figures S1B,C).

### Sequence Read Information for PBMC and UCB Samples

Sequence read information was obtained for all the captured and enriched sequence libraries constructed from 161 PBMC and 48 UCB. In PBMCs, the total draft read numbers were 281,256,904 reads with a range of reads from 398,390 to 3,196,406 reads [1,746,937 ± 457,246 standard deviation (SD) on average]. These were high-quality reads with quality values (QV) of >30 with an average QV of 35.7 ± 0.5. The draft read bases were 38.1 Gb in total with a range between 55.8 and 428.1 Mb (237.0 ± 62.6 Mb on average) and with an overall average read length of 135.6 ± 4.4 bases. In contrast, in the UCB, the total draft read numbers were 82,231,970 reads ranging from 813,198 to 7,294,988 (1,713,166 ± 900,625 on average) of high-quality reads with QV > 30 and an average QV of 36.4 ± 0.1. The total draft read bases were 11.2 Gb with a range between 111.2 and 1,007.9 Mb (232.3 ± 124.6 Mb on average) and an overall average read length of 135.5 ± 3.9 bases. Therefore, these high-quality reads had sufficient sequencing volume for further HLA genotyping analysis.

### Genotyping to the Field-3 Level for 12 HLA Loci

Nucleotide similarity searches of the sequenced HLA alleles to the field-3 level (based on synonymous and/or non-synonymous substitution in the coding region for each designated allele) using the BLAT program (50) identified a total of 177 alleles for the 161 PBMC, including 21 *HLA-A*, 39 *HLA-B*, 19 *HLA-C*, 4 *HLA-DPA1*, 16 *HLA-DPB1*, 18 *HLA-DQA1*, 16 *HLA-DQB1*, 2 *HLA-DRA*, 30 *HLA-DRB1*, 5 *HLA-DRB3*, 3 *HLA-DRB4*, and 3 *HLA-DRB5*; and 114 alleles for the 48 UCB samples including 12 *HLA-A*, 22 *HLA-B*, 14 *HLA-C*, 3 *HLA-DPA1*, 10 *HLA-DPB1*, 11 *HLA-DQA1*, 13 *HLA-DQB1*, 2 *HLA-DRA*, 18 *HLA-DRB1*, 4 *HLA-DRB3*, 3 *HLA-DRB4*, and 2 *HLA-DRB5* alleles with no phase ambiguity (Table S3). One novel allele was further detected for *HLA-DQA1* [named tentatively DQB1^*^06:02new (DDBJ Accession Number: LC499658) with a synonymous substitution in the exon 1]. In contrast, the other alleles were previously known HLA alleles that were assigned to the field-2 level at six HLA loci.

### Comparison of the RNA Levels for 12 HLA Loci and Alleles in PBMC Samples

**Figure 1:**
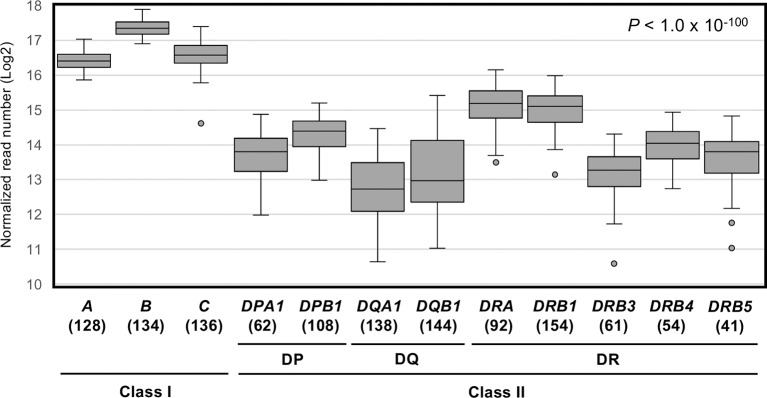
RNA expression levels of 12 human leukocyte antigen (HLA) loci in PBMC samples measured by capture RNA-Seq. These box-and-whisker diagrams were drawn using the dataset 1 obtained from the sequence reads of 78 PBMC samples (Figure S1C). Vertical axis indicates normalized read numbers (Log2) calculated as described in section Material and Methods. Horizontal axis indicates the 12 classical class I and class II HLA loci. Horizontal lines in the boxes indicate median level of expression at each locus. Parenthesis below the locus name indicates the number of individual points plotted per each locus.

Figure 1 shows a box-and-whisker diagram of the normalized read numbers (RNA levels) for 12 HLA loci using dataset 1 that includes 1,252 phased alleles identified for the PBMC samples (Table S4A). The ANOVA statistical difference between the expression levels of the HLA loci was highly significant (*P* < 1.0 × 10^−100^). We also found that the 99% confidence intervals for these 12 loci do not overlap (data not shown), confirming that each 12 loci are distinct in terms of RNA expression levels.

Among the 12 loci compared here, *HLA-B* displayed the highest average expression level. The average level of expression of *HLA-B* was ~2-fold higher than that of *HLA-A* or *HLA-C*, and ~4–5 times higher than by the class II loci, *HLA-DRA, HLA-DRB1*, or others (Table 1). In addition, the average expression levels of *HLA-DRB4* and *HLA-DRB5* were 1.4–1.5-fold higher than *HLA-DRB3* (Figure 1 and Table 1). The lowest reads were for *HLA-DQA1* and *HLA-DQB1*. The ratio of hinges (third quartile reads/first quartile reads) in the box-and-whisker diagram confirmed that locus-specific variations of the read numbers for the *HLA-DQ* RNA levels (2.8 for *HLA-DQA1* and 3.6 for *HLA-DQB1*) were much higher than 1.3–1.4 for the class I RNA levels and 1.7–2.0 for the *HLA-DP* and *HLA-DR* RNA levels (Table 1).

**Table 1:** Five-number summary of normalized RNA levels at each human leukocyte antigen (HLA) locus in peripheral blood mononuclear cell (PBMC).

**Class**	**Subregion**	**Locus**	**Minimum reads**	**First quartile reads**	**Median reads**	**Third quartile reads**	**Maximum reads**	Ratio of hinges^*^
Class I		*HLA-A*	59,565	75,996	86,788	100,109	134,318	1.3
		*HLA-B*	121,917	148,807	165,092	187,736	242,948	1.3
		*HLA-C*	56391	84,675	98,672	120,247	172,204	1.4
Class II	DP	*HLA-DPA1*	4,032	8,058	12,585	15,217	24,991	1.9
		*HLA-DPB1*	8,101	15,232	20,354	25,442	34,833	1.7
	DQ	*HLA-DQA1*	1,602	4,623	7,744	12,744	22,625	2.8
		*HLA-DQB1*	2,083	5,201	8,096	18,605	43,870	3.6
	DR	*HLA-DRA*	12,069	27,862	36,511	46,162	69,618	1.7
		*HLA-DRB1*	9,046	25,641	35,606	43,578	64,661	1.7
		*HLA-DRB3*	1,535	7,164	9,972	13,113	20,243	1.8
		*HLA-DRB4*	6,819	12,271	15,445	21,270	31,237	1.7
		*HLA-DRB5*	2,101	9,093	13,634	17,941	29,122	2.0

* *Hinges mean first and third quartiles. Ratio of hinges was calculated by third quartile reads/first quartile reads*.

**Figure 2:**
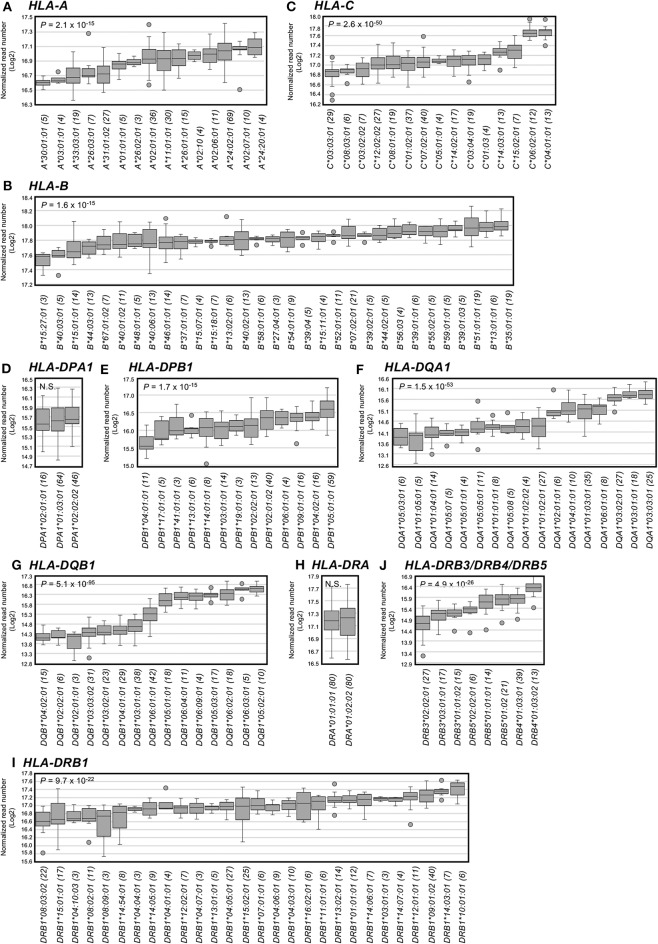
Allelic RNA levels expressed by 12 human leukocyte antigen (HLA) loci in peripheral blood mononuclear cell (PBMC) samples and measured by capture RNA-Seq. The box-and-whisker diagrams were drawn using the datasets 2–4 obtained from the sequence reads of 78 or 161 PBMC samples (Figure S1C). Panels **(A–J)** show **(A)** *HLA-A*, **(B)** *HLA-B*, **(C)** *HLA-C*, **(D)** *HLA-DPA1*, **(E)** *HLA-DPB1*, **(F)** *HLA-DQA1*, **(G)** *HLA-DQB1*, **(H)** *HLA-DRA*, **(I)** *HLA-DRB3/DRB4/DRB5*, and **(J)** *HLA-DRB1*. Vertical axis indicates normalized read numbers (log2) calculated as described in section Material and Methods. Horizontal axis indicates the alleles for each of HLA loci **(A–J)**. Horizontal lines in the box indicate median expression at each allele. Parenthesis following the allele name indicates the number of individual points plotted per each locus.

The RNA levels expressed at each allele were analyzed using the read numbers obtained from at least three different samples. Figure 2 shows box-and-whisker diagrams of the RNA levels for the HLA alleles of PBMC samples using datasets 2–4 that include 2,175 (763 class I + 1,260 class II + 152 *HLA-DRB3/DRB4/DRB5*) heterozygous alleles (Table S4A). The DAE was observed for all HLA class I and II genes, except for *HLA-DPA1* and *HLA-DRA*, with strong statistical significant differences (ANOVA) for RNA levels expressed by the HLA alleles of *HLA-B* (*P* = 2.1 × 10^−15^) to *HLA-DQB1* (*P* = 5.1 × 10^−95^) (Figures 2A–J). In addition, the ratios between the lowest and highest expressed alleles at each locus showed that *HLA-DQA1* and *HLA-DQB1* had the largest allelic differences with 3.8 (*P* = 5.0 × 10^−11^) and 5.8 (*P* = 1.4 × 10^−15^), respectively, and that there were no significant allelic differences for *HLA-DPA1* and *HLA-DRA* (Figures 2D–J and Table 2). Consistently, when degrees of DAE are compared for coefficient of variation (CV; standard deviation divided by mean), the class II *HLA-DQA1* and *HLA-DQB1* loci displayed highest levels of allelic differences compared to others, including all the class I loci, as shown in Figure S2.

**Table 2:** Comparison of the lowest and highest expressed alleles of each locus using normalized reads at median in peripheral blood mononuclear cells (PBMCs).

**Locus**	**Average median reads**	**The lowest expressed allele**		**The highest expressed allele**		Fold change^*^	***P*-value**
		**Allele**	**Median reads**	**Allele**	**Median reads**		
*HLA-A*	120,366	A^*^30:01:01	99,814	A^*^24:20:01	139,413	1.4	6.7 × 10^−4^
*HLA-B*	231,598	B^*^15:27:01	196,005	B^*^35:01:01	258,664	1.3	2.3 × 10^−6^
*HLA-C*	142,987	C^*^03:03:01	119,834	C^*^04:01:01	212,951	1.8	2.1 × 10^−18^
*HLA-DPA1*	49,804	DPA1^*^02:01:01	48,111	DPA1^*^02:02:02	51,134	1.1	NS
*HLA-DPB1*	73,344	DPB1^*^04:01:01	47,828	DPB1^*^05:01:01	99,193	2.1	1.3 × 10^−10^
*HLA-DQA1*	29,024	DQA1^*^05:03:01	14,991	DQA1^*^03:03:01	57,501	3.8	5.0 × 10^−11^
*HLA-DQB1*	50,644	DQB1^*^04:02:01	17,371	DQB1^*^05:02:01	99,929	5.8	1.4 × 10^−15^
*HLA-DRA*	151,031	DRA^*^01:01:01	148,601	DRA^*^01:02:02	153,460	1.0	NS
*HLA-DRB1*	132,803	DRB1^*^08:03:02	99,464	DRB1^*^10:01:01	182,883	1.8	6.3 × 10^−9^
*HLA-DRB3*	33,383	DRB3^*^02:02:01	26,715	DRB3^*^01:01:02	37,037	1.4	4.3 × 10^−3^
*HLA-DRB4*	70,578	DRB4^*^01:03:01	58,794	DRB4^*^01:03:02	82,362	1.4	2.5 × 10^−2^
*HLA-DRB5*	50,935	DRB5^*^02:02:01	42,508	DRB5^*^01:02	57,684	1.4	6.0 × 10^−6^

* *Fold change was calculated by reads of the highest expressed allele/reads of the lowest expressed allele*.

Based on the normalized read counts, we next compared variations among samples with the same alleles. As depicted in Figure S2, CV of read counts obtained from the samples with the same alleles at class I are relatively lower than those at class II, as the three class I loci showed the three lowest averaged CV values among all the 12 loci. It appears, therefore, that the class I genes are more evenly expressed among individuals with the same alleles, whereas class II shows more variations in expression levels among individuals with the same alleles. The larger extents of intra-allelic variations observed with the class II genes could be due to different distribution patterns of subcell populations, expressing the class II genes differently, among the samples with the same alleles.

### HLA Polymorphisms and RNA Expression Levels in Specific HLA Loci and Haplotypes of PBMC Samples

#### Relationship Between HLA-DR Haplotypes and RNA Levels

Since the correlation between *HLA-DRB1-HLA-DRB3/DRB4/DRB5* haplotypes and RNA expression levels was unknown, we examined 31 *DRB3/DRB4*, 18 *DRB3/DRB5*, and 23 *DRB4/DRB5* heterozygous samples (a total of 144 *DRB1-DRB3/DRB4/DRB5* haplotypes) from dataset 4 (Table S4A). In this dataset, there was a total of 28 *DRB1-DRB3/DRB4/DRB5* haplotypes with 14 assigned as *DRB1-DRB3*, 11 as *DRB1-DRB4*, and 3 as *DRB1-DRB5*. These haplotypes were identified by estimating *HLA-DRB1* and *HLA-DRB3/DRB4/DRB5* alleles without observing any discrepancies to previously reported HLA-DR genomic structures (53, 54).

Figure S3A shows the comparative relationships of RNA levels expressed by the *HLA-DRB1* and *HLA-DRB3/DRB4/DRB5* loci using 17 *DRB1-DRB3/DRB4/DRB5* haplotypes that were analyzed using at least three different samples (Table S5). The RNA levels expressed by *HLA-DRB1* were widely distributed for all haplotypes, whereas the expression levels of *HLA-DRB4* and *HLA-DRB5* tended to be higher than for *HLA-DRB3* (Figure S3A). In addition, there was no significant correlation between the RNA expression levels of *HLA-DRB1* and *HLA-DRB3/DRB4/DRB5* (*R*^2^ = 0.0003), indicating that they are regulated independently of each other in PBMC samples.

For RNA levels expressed by the *DRB1-DRB3/DRB4/DRB5* haplotypes (Table S5), there was a significant difference (*P* = 0.0119) between the median read numbers of DRB1^*^14:06:01/DRB3^*^02:02:01 (median, 36,822) and DRB1^*^14:54:01/DRB3^*^02:02:01 (median, 18,330). There was a significant difference (*P* = 0.0182) between DRB1^*^09:01:02 haplotypically linked to either DRB4^*^01:03:01 or DRB4^*^01:03:02 (Table S5), but no significant difference (*P* = 0.9089) between the haplotypes DRB1^*^09:01:02/ DRB4^*^01:03:01 and HLA-DRB1^*^04 or HLA-DRB1^*^07 linked to DRB4^*^01:03:01. These data show that the variance of RNA expression levels of haplotypes detected at the allelic field-2 level such as DRB1^*^09:01/DRB4^*^01:03 can be differentiated significantly at the allele field-3 level such as DRB1^*^09:01:02/ DRB4^*^01:03:01 or DRB1^*^09:01:02/ DRB4^*^01:03:01.

#### Relationships Between HLA-DQ Haplotypes and RNA Expression Levels

Since significant differences were observed for RNA levels expressed by the *HLA-DQA1* and *HLA-DQB1* alleles (Figure 2 and Table 2), we investigated the correlation between HLA-DQ haplotypes (*DQA1-DQB1*) and RNA expression levels. The distribution in the level of expression based on read numbers for the *HLA-DQA1* alleles ranged between low expression for DQA1^*^01/05 alleles, intermediate expression for DQA1^*^02/04/06 alleles, and high expression for DQA1^*^03 alleles. The DAE for *HLA-DQB1* ranged from low expression for DQB1^*^02/03/04 to high expression for DQB1^*^05/06. There were significant differences (*P* < 0.001) between most allelic groups in the low, intermediate, or high levels of expression (Figure S3B). These data were consistent with a previous report on the differential expression of *HLA-DQ* alleles in PBMC where the alleles were associated with susceptibility to and protection from type 1 diabetes (55). Interestingly, although the *DQA1-DQB1* haplotypes were composed of the highest expression group of *HLA-DQA1* alleles and the lowest expression group of *HLA-DQB1* alleles, these haplotypes occur at the highest frequency (40.5%) in the Japanese population (Figure S3B). In contrast, *DQA1-DQB1* haplotypes that are composed of highest expression groups of *HLA-DQA1* and *HLA-DQB1* alleles were not observed in the Japanese population. This finding suggests a trend for selection to low and at best intermediate expression of the DQ heterodimers. It is indeed intriguing that the highest expression groups were not observed in worldwide populations (17th IHIW: NGS HLA genotyping data, http://17ihiw.org/17th-ihiw-ngs-hla-data/) (Figure S3B).

#### RNA Expression Levels of *HLA-DPB1* Alleles and Genotypes of rs9277534

The SNP marker rs9277534 is located within the 3′UTR of *HLA-DPB1*, and the RNA levels expressed by the AA genotype were reportedly significantly (*P* < 0.001) lower than those expressed by the GG genotype (Figure S3C) (25). In our analysis of the same genotypes by the capture RNA-Seq method, the read numbers for the AA genotype using 12 samples and the GG genotype using 24 samples that were selected from dataset 3 of PBMC samples (Table S4A) produced a box-and-whisker diagram (Figure S3C) that was similar to the results of the previous report (25, 56) with a significant difference (*P* = 0.0019) of expression between the AA and GG genotypes. However, this result was not consistent with individual alleles shown in Figure 2E because DPB1^*^04:01:01 (low) and DPB1^*^05:01:01 (high) are thought to be outliers, while DPB1^*^04:02:01, DPB1^*^02:01:02 and DPB1^*^02:02 that are supposed to be low expression levels have similar RNA levels as those of DPB1^*^09:01:01 and DPB1^*^06:01:01 that are supposed to be high expression levels (57). In the case of excluding the outlier alleles, the significant difference was not obtained between the AA and GG genotypes (Figure S3C). These data suggested that not only this SNP was associated with HLA-DP expression levels but also the other (one or more SNPs) can be involved in the HLA-DP expression levels.

#### RNA Expression Levels of Null or Low Expressed HLA Alleles

Of over 25,000 HLA alleles released from the IPD-IMGT/HLA database (http://hla.alleles.org/), ~1,000 alleles are characterized as null and low expressed alleles. We tested if the RNA-Seq method is able to discriminate those null alleles by analyzing read data corresponding to the HLA-A^*^02:15N and A^*^02:53N alleles, which have been categorized as null. Examination of the RNA levels expressed by the known *HLA-A* null alleles A^*^02:15N and A^*^02:53N (Figure S3D) revealed that they were at 34.3% (41,242 reads) and 18.2% (21,902 reads), respectively, of the average median of *HLA-A* allelic expression (120,366 reads in Table 2). This result suggests that the capture RNA-Seq can directly identify null and low expression allele in a quantitative manner.

### Comparison of the HLA RNA Levels in PBMC and UCB

**Figure 3:**
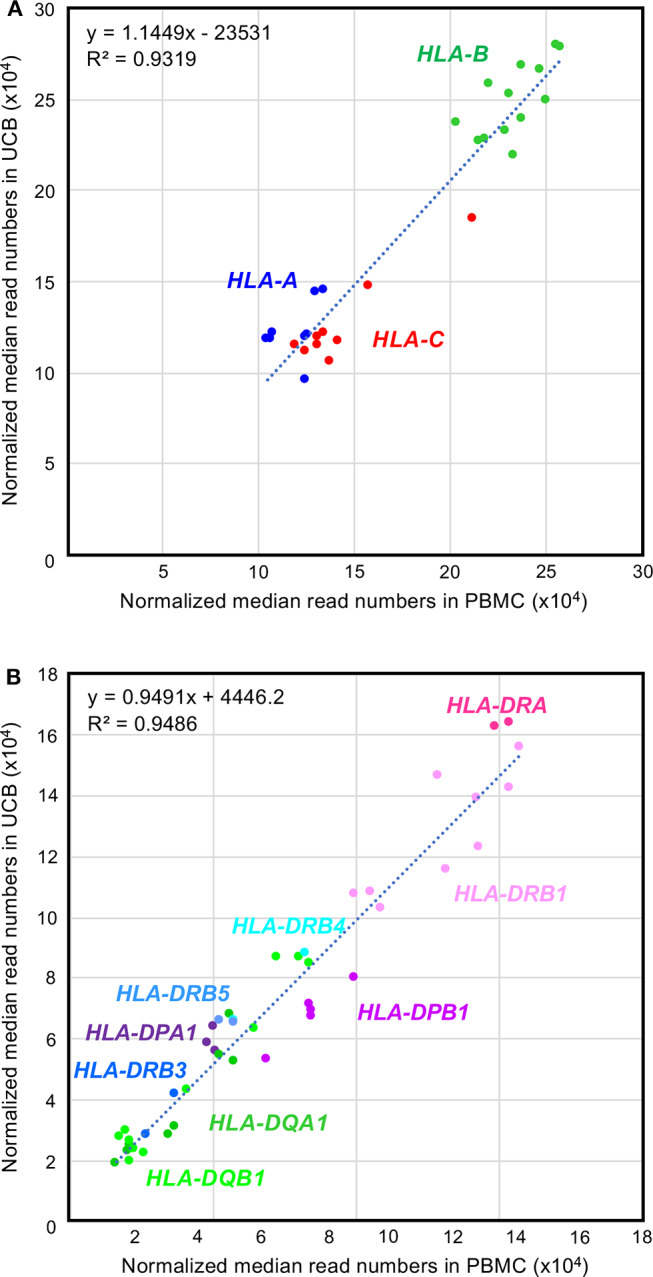
Comparison between the RNA expression levels for peripheral blood mononuclear cell (PBMC) and umbilical cord blood (UCB) samples. **(A,B)** Correlations of the RNA expression levels in the class I and class II loci, respectively. Normalized median reads (× 10^4^) in PBMC and UCB (Table S7) were plotted in the horizontal and vertical axis, respectively.

A box-and-whisker diagram of the normalized read numbers (RNA levels) expressed by 12 HLA loci (Figure S4A) in UCB was constructed using the dataset 1 that includes 280 phased alleles (Table S4B). The ANOVA statistical difference between the expression levels of the 12 HLA loci of UCB was highly significant (ANOVA, *P* < 1.0 × 10^−95^) similarly to those of PBMC (Figure 1 and Figure S4A). The DAE was observed for all HLA class I and II genes, except for *HLA-DRA* and *HLA-DRB5*. Collectively weak statistical significances were also noted for RNA levels expressed by the HLA alleles of *HLA-DPA1* (*P* = 1.2 × 10^−2^) to *HLA-DQB1* (*P* = 1.6 × 10^−31^) (Table S6). The observation of higher *p* values in UCB than in PBMC is thought to depend on the number of samples. To find differences in the RNA expression levels between the HLA alleles of PBMC and UCB, we compared 30 class I and 44 class II alleles using the normalized reads of 590 alleles (206 class I and 384 class II alleles) in UCB (Table S4B) and the corresponding 1,724 alleles (583 class I and 1,141 class II alleles) in PBMC (Table S7). Figure 3 shows a high correlation for the RNA levels expressed by HLA alleles of PBMC and UCB. The RNA levels expressed by the PBMC and UCB of HLA class I and II loci were correlated strongly with a coefficient of determination of *R*^2^ = 0.9319 and 0.9486, respectively (Figures 3A,B). However, there were significant differences between PBMC and UCB for HLA RNA levels expressed by 20 of the 74 alleles (Table S7). Most significant differences (*P* < 0.05) occurred for *HLA-A* (2 of 8 alleles), *HLA-B* (4 of 13 alleles), *HLA-C* (5 of 9 alleles), and *HLA-DPB1* (4 of 5 alleles) with no differences for 9 alleles of *HLA-DQA1* and only a few differences for the alleles of the other HLA loci. Comparatively large differences (*P* < 0.001) of RNA levels between the PBMC and UCB were observed for seven HLA alleles, A^*^11:01:01, C^*^07:02:01, DPA1^*^01:03:01, DPB1^*^05:01:01, DQB1^*^04:02:01, DRB1^*^04:05:01, and DRB1^*^01:01:01 (Figure S4B and Table S7). The results indicated that HLA expression patterns in PMBC and UCB are similar but not entirely the same with some differences. The differences are more pronounced in particular alleles, suggesting that those alleles are regulated by separated factors (genes) between PMBC and UCB.

### Validation of the Capture RNA-Seq Method

In an evaluation of our capture RNA-Seq method, Figure S5 shows the high correlation (*R*^2^ = 0.9135) that we obtained when we compared our quantitative HLA gene expression results to those previously obtained by Fagerberg et al. (58) using a RNA-Seq method to quantitate HLA gene expression levels in white blood cells (WBC) for a Human Body Map project (NCBI BioProject Accession: PRJEB2445). This comparison between different expression methods confirmed the reliability and accuracy of quantitating HLA gene expression using our capture RNA-Seq method.

We also tested to assess whether the allelic read–number differences might be generated by methodological artifacts, such as differences in probe capturing efficiencies among different alleles, by estimating allelic read-number ratios of the original quantitation targets for individual samples as well as the allelic read-number ratios of subregions of the original targets. The results, displayed in Figure S6 and showing comparisons of the allelic read number ratios of the original targets and those of their subregions for individual samples, indicated a good correlation between ratios for the original targets and those for their subregions. We, therefore, concluded that the allelic differences in the read numbers were not generated by artifacts and most likely reflect allelic differences in RNA levels. We, however, noted that small allelic differences, such as those observed with the HLA-B genes, could be derived in part from artifacts such as allelic differences in capturing efficiencies or from experimental errors.

## Discussion

In this study, we developed a protocol for genotyping 12 classical HLA genes and quantitating their gene and/or allelic expression using a single-capture RNA-Seq method and demonstrated the usefulness of the method in a comparative analysis of the RNA expression levels among the 12 HLA loci and 2,850 alleles using 161 PBMC and 48 UCB samples. For the RNA expression analysis, we chose to use previously HLA genotyped PBMC samples that had been collected as part of the Japan Marrow Donor Program (48) from medically well-tested BMT donors prior to transplantation. Therefore, these HLA-genotyped PBMC samples were from well-characterized healthy donors who were considered to be free from infection, cancer, inflammatory diseases, and other ailments that could have changed the normal baseline levels of HLA gene expression. In order to test our RNA sequencing method before applying it to disease and transplantation research, it was considered important to first understand comprehensively the normal pattern of RNA levels expressed by each allele using healthy individuals within the same ethnic group. In this way, the differences in RNA expression levels (sequence read numbers) could be compared more reliably among the HLA alleles at the different HLA loci as described previously (43).

An important aspect of our RNA-Seq method was the design and application of 172 capture RNA probe sequences of 172 most frequent Japanese HLA allele sequences that would be used to capture and enrich the targeted HLA alleles in the blood sample. If we had used only single representative allelic probes (reference probes) for each HLA gene, such as those represented by the latest version of human genome reference (e.g., GRCh38.p12 at NCBI), the target DNA fragments might not be enriched or might have been missed due to low affinity between the reference-derived RNA probe and the target HLA allele-derived DNA fragment. As a result of poor hybridization between probe and a targeted allele, the RNA expression level of a particular allele might be recorded incorrectly to be low when in fact its allelic expression was intermediate or high. Therefore, in order to minimize the false calls of a low expression, we designed the sequences of the RNA capture probes based on our previously determined HLA allele sequences in the Japanese population (49). In addition, since 93% of the 172 allele sequences cover the full length of HLA genes (5′ promoter region to 3′UTR), the 172 RNA capture probes are useful for identifying the gene sequences of disease-specific splicing variants and gene expression-related variants and for detecting antisense RNAs such as microRNAs (miRNAs) that suppress gene expression (17).

Although specific RNA capture probes are an important first step for accurate and reliable quantitation of allele expression by the capture RNA-Seq method, the NGS and analytical bioinformatic methods for obtaining correct read numbers for RNA (cDNA) sequences expressed by particular alleles are equally important steps in the overall accuracy and reliability of the protocol. In general, it is difficult to obtain the correct read numbers for each allele using the publicly available RNA-Seq analysis software because read mapping is not performed easily under 100% matching conditions, and in such cases of reduced stringency, there is a possibility that reads from other highly similar HLA genes or alleles may be mapped unexpectedly. Therefore, we focused only on coding regions (exons 2 and 3 of class I loci and exons 2 of class II loci) that can be divided easily into each polymorphic phase and the reads mapped to sequence references at 100% matching condition. Even when using our stringent mapping conditions, 72 different HLA genotypes were excluded from the subsequent analysis due to the partial mapping of the reads. In addition, to better standardize our methods, we excluded homozygous genotypes from our analysis of expressed RNA levels, although the HLA expression levels in homozygotes are approximately double those observed in heterozygotes (20). Therefore, correcting the sequence read numbers using stringent mapping conditions in our study increased the accuracy for quantitating the RNA levels expressed for each allele.

The quantitative results of RNA sequence profiles expressed by 12 HLA loci of PBMC and UCB (Figure 1 and Figure S4) in our study are consistent with the findings of others (58, 59) that the RNA levels expressed by the class I locus usually are higher than by the class II locus. While the RNA expression levels at the class I loci were in the order of *HLA-B* > *HLA-C* > *HLA-A*, the RNA expression levels at the class II locus showed lower locus-specific expression levels with the following relative order of *HLA-DRA* > *HLA-DRB1* > *HLA-DPB1* > *HLA-DPA1* > *HLA-DQB1* > *HLA-DQA1* > *HLA* = *DRB4* > *HLA* = *DRB5* > *HLA-DRB3* (Figure 1 and Table 1). The difference that we found between the different HLA loci, except for *HLA-DRB3/DRB4/DRB5*, correlated well (*R*^2^ = 0.9135) with the Illumina bodyMap2 transcriptome analysis (http://www.ensembl.info/2011/05/24/human-bodymap-2-0-data-from-illumina/) using white blood cells (NCBI BioProject Accession: PRJEB2445) (Figure S5). In addition, the RNA expression levels of the class I loci in our study were very similar to those of locus-specific real-time PCR using blood leukocytes (59). However, in the previously reported RNA-Seq, the RNA expression level at the class I loci were *HLA-A* ≈ *HLA-B* > *HLA-C*, and the RNA expression level at the class II loci were higher for the alpha chain loci than for the beta chain loci such as *HLA-DRA* > *HLA-DRB1* > *HLA-DQA1* > *HLA-DPA1* > *HLA-DQB1* > *HLA-DPB1* (43). In the RNA-Seq analysis of lymphoblastoid cell lines (LCL) (60), the RNA expression levels at the class I loci showed similar shapes with those of LCL cell lines, JY, and Pala (37). However, we found that the RNA levels expressed by some shared alleles of PBMC and UCB were significantly different (Figure S4B and Table S7). Since UCB includes stem cells and progenitor cells such as hematopoietic stem cells, mesenchymal stem cells, and vascular endothelial progenitor cells (61), the differences between PBMC and UCB for the RNA levels expressed at the same alleles probably reflect the different cell types. Our results for the RNA levels expressed by *HLA-C, HLA-DQA1, HLA-DQB1*, and *HLA-DPA1* are consistent with the results of Aguiar et al. (43). However, we also observed some inconsistencies; for example, the levels of RNA expressed by the HLA-A^*^24 alleles (A^*^24:02 and A^*^24:20) in our experiment are different from those previously obtained by quantitative PCR (qPCR) (34). This variability may reflect differences in methodology, cell types (PBMC vs. B lymphocytes), and population ethnicity. It would be important, therefore, to expand our analyses to subpopulations of cells to further understand the basis for allelic RNA-level differences.

We also noted from global comparison of the data that interallelic differences in the RNA levels are less pronounced at the *HLA-A, HLA-B*, and *HLA-C* class I loci compared to class II loci such as *HLA-DQA1* and *HLA-DQB1*. Similarly, variations among individuals with identical alleles appear to be smaller at the class I genes compared to the class II genes. Therefore, in contrast to some of the class II genes with high degrees of variations in the RNA levels, expression of the class I genes might have evolved so that their expression levels are maintained to be relatively constant among different alleles and different individuals.

Polymorphisms located in the 5′ promoter region and 3′UTR of HLA genes are known to be associated with variation in HLA expression levels (21, 32–36). Regulatory *cis* and *trans* polymorphisms that affect transcriptional regulation also are involved in susceptibility to complex, multifactorial diseases (13). In this regard, our RNA sequencing method could help to evaluate the role of polymorphisms in the *cis* and *trans* regions of the HLA genes and allele-specific regulation of HLA gene expression because our capture probes were designed so that they covered full-length HLA regions including introns and 5′ and 3′UTR regions. These polymorphic HLA UTR sequences are of interest because some of them are targets for microRNA that regulate the protein and cell surface expression of the HLA genes (17, 19, 21). The capture RNA-Seq method may provide further and more comprehensive data, which could lead to a better understanding of the molecular mechanism or polymorphisms that regulate HLA RNA expression levels. This could be developed in ways to better manage HLA-associated diseases or transplantation outcomes. For example, high expression alleles of *HLA-DPB1* were reported to be a risk effect for acute GVHD (25) (see also above), and therefore, reducing their expression levels prior to transplantation might help to reduce the risk of developing acute GVHD.

Of the 2,175 alleles at 12 HLA loci of 161 PBMC samples, we found on average that the allelic expression differences ranged from 1.3-fold for *HLA-B* to 5.8-fold for *HLA-DQB1* (Figure 2 and Table 2). Especially, large differences were observed in *HLA-DQA1* and *HLA-DQB1* loci (Figure S3B). Restrictions in DQA1/DQB1 heterodimer pairing in which DQA1^*^01 proteins (low expression) only pair with DQB1^*^05 or DQB1^*^06 proteins (high expression) and DQA1^*^03 proteins (high expression) could pair with DQB1^*^02, DQB1^*^03 and DQB1^*^04 proteins (low expression) were observed in the 17th International Histocompatibility and Immunogenetics Workshop and Workshop Conference (IHIW) NGS HLA genotyping data (http://17ihiw.org/17th-ihiw-ngs-hla-data/) (62). Therefore, it appears that the *DQA1-DQB1* linkage disequilibrium patterns could result from structural interactions of heterodimers associated with low/intermediate expression levels. It has been reported that specific allelic combinations of HLA-*DQA1, HLA-DQB1*, and *HLA-DRB1* genes influence autoimmune disease predisposition, and, furthermore, their expression levels may also correlate with causes of the disease.

In several previous studies of HLA gene expression, the HLA genes were genotyped only to the field-1 level (allele lineage level) (21, 34, 43). However, expression studies of HLA genes genotyped only to the level of an allele lineage can miss the diversity that is more evident at the field-3 level. We compared the RNA expression levels among the classified alleles at least up to the field-3 level and classified the RNA expression levels of different alleles from the same HLA locus into distinct high and low groups. For example, we classified the RNA expression levels of 15 *HLA-A* alleles for high and low groups (*P* = 1.7 × 10^−19^); A^*^26:01:01 and A^*^26:02:01 were classified into a high expression group and A^*^26:03:01 into a low expression group (Figure S3E). In *HLA-DPB1*, a strong significant difference (*P* = 7.8 × 10^−8^) was observed in the expression levels of DPB1^*^04:01 and DPB1^*^04:02. Allelic differences at the field-3 level were also observed for other HLA loci such as DQA1^*^01:03:01 vs. DQA1^*^01 group alleles, DQB1^*^06:01:01 vs. DQB1^*^06 group alleles, and DRB4^*^01:03:02 vs. DRB4^*^01:03:01 (Figure S3B and Table S5). Thus, a new nomenclature for HLA alleles that is based on DAE results might be useful, especially in the investigation of transplantation outcome, infections, autoimmunity, cancer, and drug adverse effects. In addition, DRB3^*^02:02:01 broadly linked to *HLA-DRB1* alleles and all *HLA-DRB1* types (DR3, DR11, DR12, DR13, and DR14) with an ~2-fold difference was observed in the expression levels between the DRB1^*^14:54:01 linked DRB3^*^02:02:01 and DRB1^*^14:06:01 linked DRB3^*^02:02:01 (Table S5). The current NGS methods do not fully cover all the promoter/enhancer and intronic regions, and variations in these segments could determine differences of RNA expression levels that may define the differences between the low and high expressed alleles. Therefore, the newer and most advanced NGS methods should focus on sequencing the segments that determine the RNA expression levels because these possible variations could be important for better understanding the results of associations between HLA alleles, expression, and disease.

Various quantitative methods have been used previously to study specific HLA differential allelic expression including a luciferase reporter assay (19), qPCR using locus-specific primers (21, 38), and flow cytometry using antibodies to measure cellular HLA protein expression (20, 24), but none of these methods permit interallelic comparisons. On the other hand, NGS RNA-Seq (63) and our capture RNA-Seq method enable the RNA expression levels to be compared among the different HLA alleles and enable the detection of null and low expressed HLA alleles (Figure S3D). In addition, capture RNA-Seq is a cost-saving method that allowed us to analyze 24 samples in one MiSeq run and to use the same NGS results for genotyping and for quantitating the polymorphic RNA levels. DNA polymorphism analysis of full-length HLA genes (64, 65) and HLA gene expression (58) are usually treated as separate studies. Our singular approach for genotyping RNA as cDNA and also quantitatively measuring allelic expression as sequence read numbers could be a useful new approach for evaluating molecular mechanisms that are involved in abnormal HLA gene expression in cancer cells (31) and in the pathogenesis of autoimmune diseases.

Sequencing-based whole-transcriptome analysis (i.e., RNA-Seq) is a powerful tool to measure gene expression, detect novel transcripts, characterize transcript isoforms, and identify sequence polymorphisms. Here, we described a target enrichment method where a total RNA sample was converted to a sequencing-ready cDNA library and hybridized to a large set of HLA polymorphic RNA-specific biotinylated oligonucleotide capture probes prior to NGS. The resulting sequence data were highly enriched with low expressed alleles that dramatically increased the efficiency of next-generation sequencing and the analysis of allelic expressed RNAs.

## Conclusion

This study is the first report of allele expression level differences for 12 classical HLA loci using a novel capture RNA-Seq method. The quantitative DAE data potentially provide information for predicting risks of graft rejections due to abnormally expressed HLA molecules in HCT and for discovering novel pathophysiological mechanisms in HLA-related diseases.

## Data Availability Statement

The novel HLA allele sequence is available in GenBank/DDBJ/ENBL-EBI DNA databases under the Accession Number LC499658. This work described in the article was performed with permission from The Japanese Data Center for Hematopoietic Transplantation (JDCHCT: http://www.jdchct.or.jp/en/outline/). The NGS data will be stored and maintained on a data server at Tokai University School of Medicine for at least 5 years and will be made available to interested parties upon request for validating the findings described in the paper. However, if anybody wants to use the raw NGS data beyond evaluating the current work, it is considered to be secondary usage of the data at JDCHCT (office@jdchct.or.jp), and written permission will need to be obtained from JDCHCT for such usage.

## Ethics Statement

The study protocol was approved from the institutional review board of the Japan Marrow Donor Program (JMDP) and Tokai University (Application number: 18I-48, 18I-49), and informed consents were obtained from donors in accordance with the Declaration of Helsinki. The patients/participants provided their written informed consent to participate in this study.

## Author Contributions

FY, SS, and TS participated in the design of this study. AM, AS, FY, and SI carried out most of the experiments. MM, SK, SM, YK, and YM supported the study. FY, JK, MF-V, MT, SB, SS, and TS analyzed the data and wrote the manuscript. All authors checked the final version of the paper.

## Conflict of Interest

The authors declare that the research was conducted in the absence of any commercial or financial relationships that could be construed as a potential conflict of interest.
