# Supplementary material for: Capturing Differential Allele-Level Expression and Genotypes of All Classical HLA Loci and Haplotypes by a New Capture RNA-Seq Method
Source: Front Immunol. 2020 May 29;11:941. doi: 10.3389/fimmu.2020.00941 (PMC7272581; doi:10.3389/fimmu.2020.00941)
Supplement: Supplementary file 1 [file Table_1.pdf]

Table S1. Allele frequency information for 6 HLA loci in PBMC samples

HLA-A 20 alleles (99.7%) / 62 alleles		***HLA-B*** 37 alleles (99.7%) / 103 alleles		***HLA-C*** 19 alleles (99.8%) / 47 alleles		***HLA-DRB1*** 30 alleles (99.2%) / 60 alleles		***HLA-DQB1*** 16 alleles (99.9%) / 17 alleles		***HLA-DPB1*** 16 alleles (99.8%) / 19 alleles	
Allele	Freq.	Allele	Freq.	Allele	Freq.	Allele	Freq.	Allele	Freq.	Allele	Freq.
A*24:02	36.206%	B*52:01	11.005%	C*01:02	17.262%	DRB1*09:01	14.520%	DQB1*06:01	19.080%	DPB1*05:01	38.400%
A*02:01	11.225%	B*51:01	8.728%	C*03:03	13.995%	DRB1*04:05	13.397%	DQB1*03:03	15.540%	DPB1*02:01	24.110%
A*02:06	9.496%	B*35:01	8.451%	C*07:02	12.695%	DRB1*15:02	10.248%	DQB1*04:01	12.900%	DPB1*09:01	9.950%
A*11:01	8.905%	B*15:01	7.961%	C*03:04	12.172%	DRB1*15:01	7.951%	DQB1*03:01	11.430%	DPB1*04:02	9.780%
A*31:01	8.621%	B*40:02	7.791%	C*12:02	10.875%	DRB1*08:03	7.934%	DQB1*03:02	9.590%	DPB1*04:01	5.060%
A*26:01	7.641%	B*54:01	7.586%	C*08:01	7.325%	DRB1*13:02	6.336%	DQB1*06:02	7.150%	DPB1*03:01	3.980%
A*33:03	7.370%	B*44:03	6.661%	C*14:02	6.821%	DRB1*01:01	5.629%	DQB1*05:01	6.580%	DPB1*02:02	3.410%
A*02:07	3.246%	B*40:01	5.557%	C*14:03	6.407%	DRB1*08:02	4.310%	DQB1*06:04	5.180%	DPB1*13:01	1.960%
A*26:03	2.509%	B*07:02	5.433%	C*04:01	4.337%	DRB1*12:01	3.690%	DQB1*04:02	4.210%	DPB1*14:01	1.480%
A*26:02	1.853%	B*40:06	4.787%	C*15:02	3.101%	DRB1*14:54	3.496%	DQB1*05:03	3.940%	DPB1*19:01	0.740%
A*24:20	0.770%	B*46:01	4.505%	C*08:03	1.460%	DRB1*04:06	3.302%	DQB1*05:02	2.640%	DPB1*06:01	0.570%
A*01:01	0.440%	B*39:01	3.395%	C*07:04	1.000%	DRB1*04:03	3.142%	DQB1*06:03	0.600%	DPB1*17:01	0.140%
A*03:01	0.436%	B*48:01	2.890%	C*06:02	0.822%	DRB1*11:01	2.496%	DQB1*06:09	0.570%	DPB1*41:01	0.100%
A*02:10	0.433%	B*55:02	2.470%	C*03:02	0.669%	DRB1*14:05	2.144%	DQB1*02:02	0.370%	DPB1*38:01	0.070%
A*30:01	0.179%	B*59:01	2.032%	C*05:01	0.401%	DRB1*04:10	2.127%	DQB1*02:01	0.130%	DPB1*47:01	0.030%
A*11:02	0.163%	B*15:18	1.581%	C*01:03	0.340%	DRB1*12:02	1.672%	DQB1*06:22	Unknown	DPB1*48:01	Unknown
A*03:02	0.080%	B*13:01	1.166%	C*12:03	0.094%	DRB1*14:03	1.625%				
A*02:18	0.061%	B*67:01	1.133%	C*03:23	0.023%	DRB1*14:06	1.561%				
A*02:15N	0.008%	B*15:11	0.943%	C*08:22	0.001%	DRB1*04:01	1.041%				
A*02:53N	0.008%	B*56:01	0.914%			DRB1*13:01	0.580%				
		B*58:01	0.661%			DRB1*04:07	0.503%				
		B*15:07	0.619%			DRB1*10:01	0.473%				
		B*37:01	0.514%			DRB1*07:01	0.357%				
		B*40:03	0.435%			DRB1*04:04	0.194%				
		B*44:02	0.417%			DRB1*16:02	0.183%				
		B*39:02	0.302%			DRB1*03:01	0.137%				
		B*13:02	0.277%			DRB1*14:07	0.105%				
		B*38:02	0.265%			DRB1*08:09	0.045%				
		B*39:04	0.226%			DRB1*14:02	0.027%				
		B*51:02	0.222%			DRB1*13:07	0.021%				
		B*27:04	0.205%								
		B*56:03	0.182%								
		B*55:04	0.152%								
		B*15:27	0.106%								
		B*27:05	0.065%								
		B*39:23	0.030%								
		B*40:50	0.013%								

Percentage and allele numbers indicate cumulative frequency of HLA alleles and total allele numbers analyzed in this study, respectively. Square indicates HLA alleles that have allele frequency over 0.05% in Japanese population. The HLA allele frequency data is released by HLA laboratory webpage (http:/http://www.hla.or.jp/haplo/haplonavi).
