# Supplementary material for: Capturing Differential Allele-Level Expression and Genotypes of All Classical HLA Loci and Haplotypes by a New Capture RNA-Seq Method
Source: Front Immunol. 2020 May 29;11:941. doi: 10.3389/fimmu.2020.00941 (PMC7272581; doi:10.3389/fimmu.2020.00941)
Supplement: Supplementary file 2 [file Table_2.pdf]

Table S2. 172 HLA allele information used for the design of sequence capture probes

HLA-A (19 sequences)		
Allele name	Accession number	Covered region
*A*01:01:01:01*	LC257682	Full region
*A*02:01:01:01*	LC257683	Full region
*A*02:03:01*	LC257684	Full region
*A*02:06:01:01*	LC257685	Full region
*A*02:07:01*	LC257686	Full region
*A*02:10*	LC257687	Full region
*A*02:18*	LC257688	Full region
*A*03:01:01:01*	LC257689	Full region
*A*03:02:01*	LC257690	Full region
*A*11:01:01:01*	LC257691	Full region
*A*11:02:01*	LC257692	Full region
*A*24:02:01:01*	LC257693	Full region
*A*24:20:01:01*	LC257694	Full region
*A*26:01:01:01*	LC257696	Full region
*A*26:02:01*	LC257697	Full region
*A*26:03:01*	LC257698	Full region
*A*30:01:01*	LC257699	Full region
*A*31:01:02:01*	LC257700	Full region
*A*33:03:01*	LC257701	Full region

HLA-B (39 sequences)		
Allele name	Accession number	Covered region
*B*07:02:01:01*	LC257702	Full region
*B*13:01:01:01*	LC257704	Full region
*B*13:02:01*	LC257705	Full region
*B*15:01:01:01*	LC257706	Full region
*B*15:02:01*	LC257707	Full region
*B*15:07:01:01*	LC257708	Full region
*B*15:11:01*	LC257709	Full region
*B*15:18:01:02*	LC257710	Full region
*B*15:27:01*	LC257711	Full region
*B*15:428*	LC257712	Full region
*B*27:04:01*	LC257713	Full region
*B*27:05:02:05*	LC257714	Full region
*B*35:01:01:02*	LC257715	Full region
*B*37:01:01:01*	LC257716	Full region
*B*38:02:01*	LC257717	Full region
*B*39:01:01:03*	LC257718	Full region
*B*39:02:03*	LC257721	Full region
*B*39:04*	LC257722	Full region
*B*39:23*	LC257723	Full region
*B*40:01:02:01/04*	LC257724	Full region
*B*40:02:01:01*	LC257726	Full region
*B*40:03:01:02*	LC257727	Full region
*B*40:06:01:01*	LC257728	Full region
*B*40:50*	LC257729	Full region
*B*44:02:01:01*	LC257730	Full region
*B*44:03:01:10*	LC257731	Full region
*B*46:01:01*	LC257732	Full region
*B*48:01:01:01*	LC257733	Full region
*B*51:01:01:01*	LC257735	Full region
*B*51:02:01:01*	LC257737	Full region
*B*52:01:01:02*	LC257738	Full region
*B*54:01:01*	LC257739	Full region
*B*55:02:01:03*	LC257740	Full region
*B*55:04*	LC257741	Full region
*B*56:01:01:03*	LC257742	Full region
*B*56:03*	LC257743	Full region
*B*58:01:01:03*	LC257744	Full region
*B*59:01:01:02*	LC257745	Full region
*B*67:01:02*	LC257746	Full region

HLA-C (19 sequences)		
Allele name	Accession number	Covered region
*C*01:02:01:01*	LC257747	Full region
*C*01:03*	LC257749	Full region
*C*03:02:02:01*	LC257750	Full region
*C*03:03:01:01*	LC257751	Full region
*C*03:04:01:01*	LC257754	Full region
*C*03:23*	AB618605	Exons 2, 3 and 4
*C*04:01:01:01*	LC257756	Full region
*C*05:01:01:02*	LC257757	Full region
*C*06:02:01:01*	LC257758	Full region
*C*07:02:01:01*	LC257759	Full region
*C*07:04:01:01*	LC257763	Full region
*C*08:01:01:01*	LC257764	Full region
*C*08:03:01*	LC257766	Full region
*C*08:22*	LC257767	Full region
*C*12:02:02:01*	LC257768	Full region
*C*12:03:01:01*	LC257769	Full region
*C*14:02:01:01*	LC257770	Full region
*C*14:03*	LC257771	Full region
*C*15:02:01:01*	LC257772	Full region

HLA-DRA (2 sequences)		
Allele name	Accession number	Covered region
*DRA*01:01:01:01*	AL935032	Full region
*DRA*01:02:02:01*	AL670296	Full region

HLA-DRB1 (31 sequences)		
Allele name	Accession number	Covered region
*DRB1*01:01:01*	LC257773	Full region
*DRB1*03:01:01:03*	LC257774	Full region
*DRB1*04:01:01:03*	LC257775	Full region
*DRB1*04:03:01:02*	LC257776	Full region
*DRB1*04:04:01*	LC257777	Full region
*DRB1*04:05:01:01*	LC257778	Full region
*DRB1*04:06:01*	LC257781	Full region
*DRB1*04:07:01:02*	LC257782	Full region
*DRB1*04:10:03*	LC257783	Full region
*DRB1*07:01:01:01*	LC257784	Full region
*DRB1*08:02:01:01*	LC257785	Full region
*DRB1*08:03:02:02*	LC257787	Full region
*DRB1*08:09*	LC257788	Full region
*DRB1*09:01:02:01*	LC257789	Full region
*DRB1*10:01:01:03*	LC257791	Full region
*DRB1*11:01:01:03*	LC257792	Full region
*DRB1*11:01:01:04*	LC257793	Full region
*DRB1*12:01:01:04*	LC257794	Full region
*DRB1*12:02:01:03*	LC257797	Full region
*DRB1*13:01:01:01*	LC257799	Full region
*DRB1*13:02:01:02*	LC257800	Full region
*DRB1*13:07:01*	LC257802	Full region
*DRB1*14:02:01:02*	LC257803	Full region
*DRB1*14:03:01*	LC257804	Full region
*DRB1*14:05:01:02*	LC257805	Full region
*DRB1*14:06:01*	LC257806	Full region
*DRB1*14:07:01*	LC257807	Full region
*DRB1*14:54:01:02*	LC257809	Full region
*DRB1*15:01:01:03*	LC257810	Full region
*DRB1*15:02:01:03*	LC257812	Full region
*DRB1*16:02:01:03*	LC257813	Full region

HLA-DRB3/DRB4/DRB5 (9 sequences)		
Allele name	Accession number	Covered region
*DRB3*01:01:02:01*	KX687284	Full region
*DRB3*02:02:01:01*	AL929581	Full region
*DRB3*03:01:01*	AB829532	Exons 2 - 3'UTR
*DRB4*01:01:01:01*	BX927235	Full region
*DRB4*01:03:01:01*	CR788250	Full region
*DRB4*01:03:02*	AB829536	Exons 2 - 3'UTR
*DRB5*01:01:01*	AB829537	Exons 2 - 3'UTR
*DRB5*01:02*	AB829538	Exons 2 - 3'UTR
*DRB5*02:02:01*	AB829539	Exons 2 - 3'UTR

HLA-DQA1 (16 sequences)		
Allele name	Accession number	Covered region
*DQA1*01:01:01:04*	LC257814	Full region
*DQA1*01:02:01:01*	LC257816	Full region
*DQA1*01:03:01:01*	LC257821	Full region
*DQA1*01:04:01:01*	LC257825	Full region
*DQA1*01:05:01*	LC257827	Full region
*DQA1*02:01:01:01*	LC257828	Full region
*DQA1*03:01:01*	LC257829	Full region
*DQA1*03:02:01:01*	LC257830	Full region
*DQA1*03:03:01:01*	LC257833	Full region
*DQA1*04:01:01:01*	LC257837	Full region
*DQA1*05:01:01:03*	LC257840	Full region
*DQA1*05:03:01:02*	LC257841	Full region
*DQA1*05:05:01:03*	LC257842	Full region
*DQA1*05:06:01:01*	LC257845	Full region
*DQA1*05:07*	LC257847	Full region
*DQA1*06:01:01:01*	LC257848	Full region

HLA-DQB1 (15 sequences)		
Allele name	Accession number	Covered region
*DQB1*02:01:01*	LC257850	Full region
*DQB1*02:02:01:01*	LC257851	Full region
*DQB1*03:01:01:01*	LC257852	Full region
*DQB1*03:02:01:01*	LC257859	Full region
*DQB1*03:03:02:02/03*	LC257860	Full region
*DQB1*04:01:01:02*	LC257861	Full region
*DQB1*04:02:01:04*	LC257862	Full region
*DQB1*05:01:01:03*	LC257865	Full region
*DQB1*05:02:01:02*	LC257867	Full region
*DQB1*05:03:01:01*	LC257869	Full region
*DQB1*06:01:01:01*	LC257872	Full region
*DQB1*06:02:01:01*	LC257874	Full region
*DQB1*06:03:01:01*	LC257875	Full region
*DQB1*06:04:01*	LC257876	Full region
*DQB1*06:09:01:01*	LC257877	Full region
*DQB1*06:22*		

HLA-DPA1 (4 sequences)		
Allele name	Accession number	Covered region
*DPA1*01:03:01:05*	LC257881	Full region
*DPA1*02:01:01:01*	LC257886	Full region
*DPA1*02:02:02:01*	LC257889	Full region
*DPA1*02:07:01:01*	LC257892	Full region

HLA-DPB1 (18 sequencces)		
Allele name	Accession number	Covered region
*DPB1*02:01:02:01*	LC257894	Full region
*DPB1*02:02:01:05*	LC257902	Full region
*DPB1*03:01:01:01*	LC257905	Full region
*DPB1*04:01:01:01*	LC257908	Full region
*DPB1*04:02:01:02*	LC257910	Full region
*DPB1*05:01:01:01*	LC257912	Full region
*DPB1*06:01:01:01*	LC257920	Full region
*DPB1*09:01:01*	LC257921	Full region
*DPB1*13:01:01:05*	LC257923	Full region
*DPB1*14:01:01:01*	LC257924	Full region
*DPB1*17:01:01:01*	LC257925	Exons 2 - 3'UTR
*DPB1*19:01:01:01*	LC257926	Full region
*DPB1*25:01*	LC257928	Exons 2 - 3'UTR
*DPB1*36:01*	LC257929	Exons 2 - 3'UTR
*DPB1*38:01*	LC257930	Exons 2 - 3'UTR
*DPB1*41:01:01:01*	LC257931	Exons 2 - 3'UTR
*DPB1*47:01:01:01*	LC257932	Full region
*DPB1*48:01*	LC257933	Exon 2
