# Supplementary material for: Capturing Differential Allele-Level Expression and Genotypes of All Classical HLA Loci and Haplotypes by a New Capture RNA-Seq Method
Source: Front Immunol. 2020 May 29;11:941. doi: 10.3389/fimmu.2020.00941 (PMC7272581; doi:10.3389/fimmu.2020.00941)
Supplement: Supplementary file 3 [file Table_3.pdf]

Table S3. Assigned allele names and numbers in PBMC and UCB

HLA-A			*HLA-B*			*HLA-C*		
Allele	PBMC	UCB	Allele	PBMC	UCB	Allele	PBMC	UCB
*A*01:01:01*	7	1	*B*07:02:01*	11	7	*C*01:02:01*	50	15
*A*02:01:01*	44	6	*B*13:01:01*	6		*C*01:03*	6	
*A*02:06:01*	15	9	*B*13:02:01*	6		*C*03:02:02*	7	3
*A*02:07:01*	10	2	*B*15:01:01*	20	10	*C*03:03:01*	39	14
*A*02:10*	5		*B*15:07:01*	6	1	*C*03:04:01*	27	11
*A*02:15N*	1		*B*15:11:01*	5		*C*03:23*	3	
*A*02:18*	2		*B*15:18:01*	8		*C*04:01:01*	16	7
*A*02:53N*	1		*B*15:27:01*	4		*C*05:01:01*	5	
*A*03:01:01*	4		*B*27:04:01*	3		*C*06:02:01*	15	
*A*03:01:04*	1		*B*27:05:02*	1		*C*07:02:01*	49	19
*A*03:02:01*	1		*B*35:01:01*	21	7	*C*07:04:01*	5	1
*A*11:01:01*	35	6	*B*37:01:01*	9		*C*08:01:01*	23	4
*A*11:02:01*	2		*B*38:02:01*	2	2	*C*08:03:01*	6	
*A*24:02:01*	95	35	*B*39:01:01*	5	4	*C*08:22:01*	2	1
*A*24:20:01*	5	1	*B*39:01:03*	5	2	*C*12:02:02*	29	7
*A*26:01:01*	20	5	*B*39:02:01*	5	2	*C*12:03:01*	1	1
*A*26:02:01*	5	1	*B*39:02:03*	2		*C*14:02:01*	17	4
*A*26:03:01*	9	7	*B*39:04*	5		*C*14:03:01*	15	7
*A*30:01:01*	5		*B*39:23*	1		*C*15:02:01*	7	2
*A*31:01:02*	32	15	*B*40:01:02*	13	8			
*A*33:03:01*	23	8	*B*40:02:01*	17	8			
			*B*40:03:01*	5				
			*B*40:06:01*	15	3			
			*B*40:50:01*	1				
			*B*44:02:01*	5				
			*B*44:03:01*	14	7			
			*B*46:01:01*	21	3			
			*B*48:01:01*	6	2			
			*B*51:01:01*	21	7			
			*B*51:02:01*	2				
			*B*52:01:01*	26	8			
			*B*54:01:01*	12	3			
			*B*55:02:01*	8	6			
			*B*55:04*	1				
			*B*56:01:01*	5	1			
			*B*56:03*	5	1			
			*B*58:01:01*	7	3			
			*B*59:01:01*	6	1			
			*B*67:01:01*	7				
21	322	96	39	322	96	19	322	96

HLA-DPA1			*HLA-DPB1*			*HLA-DQA1*		
Allele	PBMC	UCB	Allele	PBMC	UCB	Allele	PBMC	UCB
*DPA1*01:03:01*	130	33	*DPB1*02:01:02*	67	22	*DQA1*01:01:01*	14	8
*DPA1*02:01:01*	47	15	*DPB1*02:02:01*	22	4	*DQA1*01:02:01*	36	21
*DPA1*02:02:02*	138	48	*DPB1*03:01:01*	16	2	*DQA1*01:02:02*	4	
*DPA1*02:07:01*	7		*DPB1*04:01:01*	11	2	*DQA1*01:03:01*	54	12
			*DPB1*04:02:01*	26	9	*DQA1*01:04:01*	21	5
			*DPB1*05:01:01*	118	45	*DQA1*01:05:01*	6	
			*DPB1*06:01:01*	4		*DQA1*02:01:01*	6	
			*DPB1*09:01:01*	18	6	*DQA1*03:01:01*	34	7
			*DPB1*13:01:01*	6	2	*DQA1*03:02:01*	44	17
			*DPB1*14:01:01*	11	3	*DQA1*03:03:01*	46	10
			*DPB1*17:01:01*	6		*DQA1*04:01:01*	11	7
			*DPB1*19:01:01*	5		*DQA1*05:01:01*	4	
			*DPB1*38:01*	4		*DQA1*05:03:01*	9	2
			*DPB1*41:01:01*	4		*DQA1*05:05:01*	11	6
			*DPB1*47:01:01*	3		*DQA1*05:06:01*	2	
			*DPB1*48:01*	1		*DQA1*05:07*	5	
			*DPB1*104:01:01*		1	*DQA1*05:08*	6	
						*DQA1*06:01:01*	9	1
4	322	96	17	322	96	18	322	96

HLA-DQB1			*HLA-DRA*			*HLA-DRB1*		
Allele	PBMC	UCB	Allele	PBMC	UCB	Allele	PBMC	UCB
*DQB1*02:01:01*	3		*DRA*01:01:01*	198	60	*DRB1*01:01:01*	12	8
*DQB1*02:02:01*	6		*DRA*01:02:02*	124	36	*DRB1*03:01:01*	3	
*DQB1*03:01:01*	49	11				*DRB1*04:01:01*	5	1
*DQB1*03:02:01*	34	7				*DRB1*04:03:01*	13	2
*DQB1*03:03:02*	43	16				*DRB1*04:04:01*	5	
*DQB1*04:01:01*	36	10				*DRB1*04:05:01*	36	10
*DQB1*04:02:01*	16	6				*DRB1*04:06:01*	9	2
*DQB1*05:01:01*	20	8				*DRB1*04:07:01*	4	
*DQB1*05:02:01*	10	1				*DRB1*04:10:03*	5	
*DQB1*05:03:01*	17	4				*DRB1*07:01:01*	6	
*DQB1*06:01:01*	48	12				*DRB1*08:02:01*	11	8
*DQB1*06:02:01*	18	11				*DRB1*08:03:02*	26	4
*DQB1*06:02:new*		1				*DRB1*08:09:01*	3	1
*DQB1*06:03:01*	5					*DRB1*09:01:02*	44	16
*DQB1*06:04:01*	11	7				*DRB1*10:01:01*	8	
*DQB1*06:09:01*	5	2				*DRB1*11:01:01*	6	2
*DQB1*06:22:03*	1					*DRB1*12:01:01*	12	4
						*DRB1*12:02:01*	8	
						*DRB1*13:01:01*	5	
						*DRB1*13:02:01*	16	9
						*DRB1*13:07:01*	1	
						*DRB1*14:02:01*	1	
						*DRB1*14:03:01*	7	1
						*DRB1*14:05:01*	9	3
						*DRB1*14:06:01*	7	1
						*DRB1*14:07:01*	4	
						*DRB1*14:54:01*	8	2
						*DRB1*15:01:01*	18	13
						*DRB1*15:02:01*	24	9
						*DRB1*16:02:01*	6	
17	322	96	2	322	96	30	322	96

HLA-DRB3			*HLA-DRB4*			*HLA-DRB5*		
Allele	PBMC	UCB	Allele	PBMC	UCB	Allele	PBMC	UCB
*DRB3*01:01:02*	22	4	*DRB4*01:02*	5	1	*DRB5*01:01:01*	18	13
*DRB3*01:01:05*	2	1	*DRB4*01:03:01*	85	14	*DRB5*01:02*	24	9
*DRB3*02:02:01*	40	8	*DRB4*01:03:02*	37	16	*DRB5*02:02:01*	6	
*DRB3*03:01:01*	16	9						
*DRB3*03:01:03*	7							
5	87	22	3	127	31	3	48	22

Red letter indicates a novel allele.
