# Supplementary material for: Capturing Differential Allele-Level Expression and Genotypes of All Classical HLA Loci and Haplotypes by a New Capture RNA-Seq Method
Source: Front Immunol. 2020 May 29;11:941. doi: 10.3389/fimmu.2020.00941 (PMC7272581; doi:10.3389/fimmu.2020.00941)
Supplement: Supplementary file 4 [file Table_4.pdf]

Table S4. Information for analyzed and excluded alleles identified at 12 HLA loci by capture RNA-seq

A. Classification for analyzed and excluded allele numbers in 161 PBMC

Dataset	Locus	Analyzed allele number			Excluded allele number			Total
		Total	3 or more	n<3	Homozygote	Partially phased	Hemizygote	
1	HLA 12 loci	1,252	/	/	200	108	0	1,560
2	*HLA-A*	254	249	5	38	30	0	322
	HLA-B	272	260	12	6	44	0	322
	HLA-C	258	254	4	28	36	0	322
	Total	784	763	21	72	110	0	966
3	*HLA-DPA1*	128	126	2	156	38	0	322
	HLA-DPB1	200	198	2	78	44	0	322
	HLA-DQA1	220	218	2	34	68	0	322
	HLA-DQB1	270	270	0	32	20	0	322
	HLA-DRA	160	160	0	162	0	0	322
	HLA-DRB1	290	288	2	18	14	0	322
	Total	1,268	1,260	8	480	184	0	1,932
4	Dataset 3 + *HLA-DRB3/DRB4/DRB5*	156	152	4	56	0	50	262

B. Classification for analyzed and excluded allele numbers in 48 UCB

Dataset	Locus	Analyzed allele number			Excluded allele number			Total
		Total	3 or more	n<3	Homozygote	Partially phased	Hemizygote	
1	HLA 12 loci	280	/	/	54	26	0	360
2	*HLA-A*	68	64	4	10	18	0	96
	HLA-B	86	75	11	4	6	0	96
	HLA-C	74	67	7	14	8	0	96
	Total	228	206	22	28	32	0	288
3	*HLA-DPA1*	50	50	0	38	8	0	96
	HLA-DPB1	60	53	7	30	6	0	96
	HLA-DQA1	54	51	3	14	28	0	96
	HLA-DQB1	80	77	3	8	8	0	96
	HLA-DRA	48	48	0	48	0	0	96
	HLA-DRB1	86	71	15	8	2	0	96
	Total	378	350	28	146	52	0	576
4	Dataset 3 + *HLA-DRB3/DRB4/DRB5*	36	34	2	22	0	17	75

The application of the dataset was shown in Figure S1C.
