# Supplementary material for: Capturing Differential Allele-Level Expression and Genotypes of All Classical HLA Loci and Haplotypes by a New Capture RNA-Seq Method
Source: Front Immunol. 2020 May 29;11:941. doi: 10.3389/fimmu.2020.00941 (PMC7272581; doi:10.3389/fimmu.2020.00941)
Supplement: Supplementary file 5 [file Table_5.pdf]

Table S5. *DRB1-DRB3/DRB4/DRB5* haplotypes and their median read numbers

DR haplotype	DR type	*DRB1* type	DRB1 allele name	*DRB3/DRB4/DRB5* allele name	Haplotype number	Reads at median in *DRB1*	Reads at median in *DRB3/DRB4/DRB5*
DR52	DR3	DR3	*DRB1*03:01:01*	*DRB3*02:02:01*	1	140,371	17,026
	DR5	DR11	*DRB1*11:01:01*	*DRB3*02:02:01*	2	122,262	22,171
		DR12	*DRB1*12:01:01*	*DRB3*01:01:02*	5	134,929	39,881
			*DRB1*12:01:01*	*DRB3*01:01:05*	2	143,183	42,094
			*DRB1*12:02:01*	*DRB3*03:01:03*	2	106,214	24,142
			*DRB1*12:02:01*	*DRB3*02:02:01*	1	134,672	29,160
	DR6	DR13	*DRB1*13:01:01*	*DRB3*01:01:02*	3	115,346	32,855
			*DRB1*13:02:01*	*DRB3*03:01:01*	10	125,640	39,785
			*DRB1*13:07:01*	*DRB3*02:02:01*	1	146,940	43,446
		DR14	*DRB1*14:03:01*	*DRB3*01:01:02*	5	147,258	34,972
			*DRB1*14:05:01*	*DRB3*02:02:01*	5	111,483	26,177
			*DRB1*14:06:01*	*DRB3*02:02:01*	4	128,564	36,822
			*DRB1*14:07:01*	*DRB3*02:02:01*	2	130,309	33,453
			*DRB1*14:54:01*	*DRB3*02:02:01*	6	107,240	18,330
DR53	DR4	DR4	*DRB1*04:01:01*	*DRB4*01:02*	2	113,101	50,486
			*DRB1*04:03:01*	*DRB4*01:03:01*	7	112,638	60,666
			*DRB1*04:04:01*	*DRB4*01:03:01*	3	109,306	58,403
			*DRB1*04:05:01*	*DRB4*01:03:01*	15	116,002	54,489
			*DRB1*04:05:01*	*DRB4*01:03:02*	1	117,672	43,797
			*DRB1*04:06:01*	*DRB4*01:03:01*	2	120,304	53,462
			*DRB1*04:07:01*	*DRB4*01:03:01*	2	114,593	62,977
			*DRB1*04:10:03*	*DRB4*01:03:01*	3	97,589	65,618
	DR7	DR7	*DRB1*07:01:01*	*DRB4*01:03:01*	3	108,221	62,668
	DR9	DR9	*DRB1*09:01:02*	*DRB4*01:03:02*	12	136,632	82,865
			*DRB1*09:01:02*	*DRB4*01:03:01*	4	142,761	59,098
DR51	DR2	DR15	*DRB1*15:01:01*	*DRB5*01:01:01*	14	104,271	52,615
			*DRB1*15:02:01*	*DRB5*01:02*	21	121,102	57,684
		DR16	*DRB1*16:02:01*	*DRB5*02:02:01*	6	129,030	42,508

Gray background indicates less than 3 haplotype numbers.
