# Supplementary material for: Capturing Differential Allele-Level Expression and Genotypes of All Classical HLA Loci and Haplotypes by a New Capture RNA-Seq Method
Source: Front Immunol. 2020 May 29;11:941. doi: 10.3389/fimmu.2020.00941 (PMC7272581; doi:10.3389/fimmu.2020.00941)
Supplement: Supplementary file 6 [file Table_6.pdf]

Table S6. Comparison of the lowest and highest expressed alleles of each locus using normalized reads at median in UCBs

Locus	Average median reads	The lowest expressed allele		The highest expressed allele		Fold-change*	*P* -value
		Allele	Median reads	Allele	Median reads		
HLA-A	122,782	*A*11:01:01*	95,937	*A*24:02:01*	145,088	1.5	1.1×10^{-9}
HLA-B	248,836	*B*54:01:01*	219,328	*B*51:01:01*	248,836	1.1	1.1×10^{-10}
HLA-C	126,571	*C*07:02:01*	105,541	*C*04:01:01*	184,177	1.7	2.8×10^{-20}
HLA-DPA1	59,286	*DPA1*02:02:02*	55,594	*DPA1*01:03:01*	63,753	1.1	1.2×10^{-2}
HLA-DPB1	68,176	*DPB1*14:01:01*	53,029	*DPB1*05:01:01*	80,023	1.5	6.1×10^{-4}
HLA-DQA1	36,700	*DQA1*01:04:01*	19,044	*DQA1*03:01:01*	67,992	3.6	8.6×10^{-10}
HLA-DQB1	48,330	*DQB1*03:03:02*	19,194	*DQB1*05:03:01*	86,679	4.5	1.6×10^{-31}
HLA-DRA	163,429	*DRA*01:01:01*	162,713	*DRA*01:02:02*	164,146	1.0	NS
HLA-DRB1	126,921	*DRB1*15:01:01*	102,839	*DRB1*09:01:02*	155,997	1.5	4.0×10^{-13}
HLA-DRB3	34,934	*DRB3*02:02:01*	28,528	*DRB3*03:01:01*	41,339	1.4	2.6×10^{-3}
HLA-DRB4	76,943	*DRB4*01:03:01*	66,047	*DRB4*01:03:02*	87,839	1.3	3.6×10^{-3}
HLA-DRB5	65,245	*DRB5*01:02*	64,935	*DRB5*01:01:01*	65,555	1.0	NS

*Fold-change was calculated by reads of the highest expressed allele / reads of the lowest expressed allele.
