# Supplementary material for: Capturing Differential Allele-Level Expression and Genotypes of All Classical HLA Loci and Haplotypes by a New Capture RNA-Seq Method
Source: Front Immunol. 2020 May 29;11:941. doi: 10.3389/fimmu.2020.00941 (PMC7272581; doi:10.3389/fimmu.2020.00941)
Supplement: Supplementary file 7 [file Table_7.pdf]

Table S7. Average median of the read numbers expressed by alleles at 12 HLA loci of PBMC and UCB samples

Locus	Allele	PBMCs		UCBs		*P* -value
		Sample number	Average median	Sample number	Average median	
HLA-A	*A*33:03:01*	19	104,595	8	118,605	NS
	*A*26:03:01*	7	106,407	5	117,781	NS
	*A*31:01:02*	27	108,249	9	121,485	NS
	*A*02:01:01*	36	124,661	3	119,346	NS
	*A*11:01:01*	30	124,881	5	95,937	8.6×10^{-4}
	*A*26:01:01*	15	125,479	4	120,159	NS
	*A*02:06:01*	11	130,508	6	143,852	NS
	*A*24:02:01*	69	134,977	24	145,088	1.7×10^{-2}
HLA-B	*B*15:01:01*	14	204,121	8	237,078	NS
	*B*44:03:01*	13	215,717	7	226,642	2.9×10^{-2}
	*B*40:01:02*	11	219,006	7	228,199	NS
	*B*40:06:01*	13	220,635	3	258,239	3.2×10^{-2}
	*B*40:02:01*	13	229,257	7	232,489	NS
	*B*58:01:01*	6	231,149	3	252,292	6.6×10^{-3}
	*B*54:01:01*	9	233,887	3	219,328	NS
	*B*52:01:01*	11	237,498	7	239,487	NS
	*B*55:02:01*	21	238,360	7	268,450	6.3×10^{-3}
	*B*07:02:01*	6	247,467	6	265,642	NS
	*B*39:01:01*	10	250,429	4	249,636	NS
	*B*51:01:01*	19	255,826	7	278,880	NS
	*B*35:01:01*	19	258,664	6	278,508	NS
HLA-C	*C*03:03:01*	29	119,624	10	114,418	NS
	*C*03:02:02*	7	124,624	3	111,778	NS
	*C*12:02:02*	27	131,286	7	119,288	1.3×10^{-2}
	*C*08:01:01*	19	131,722	4	117,658	NS
	*C*01:02:01*	37	134,331	13	121,071	5.0×10^{-3}
	*C*07:02:01*	40	137,165	9	105,541	1.0×10^{-7}
	*C*03:04:01*	19	141,832	9	117,564	6.2×10^{-3}
	*C*14:03:01*	13	157,490	6	147,641	NS
	*C*04:01:01*	13	212,866	6	184,177	4.1×10^{-2}
HLA-DPA1	*DPA1*02:01:01*	16	48,111	9	58,510	NS
	*DPA1*01:03:01*	64	50,166	25	63,753	4.9×10^{-4}
	*DPA1*02:02:02*	46	51,134	16	55,594	NS
HLA-DPB1	*DPB1*14:01:01*	8	68,766	3	53,029	NS
	*DPB1*02:01:02*	40	83,570	19	71,434	2.5×10^{-3}
	*DPB1*09:01:01*	16	84,146	6	67,388	2.1×10^{-2}
	*DPB1*04:02:01*	16	84,727	6	69,005	8.0×10^{-3}
	*DPB1*05:01:01*	59	99,193	19	80,023	3.7×10^{-4}
HLA-DQA1	*DQA1*01:04:01*	14	16,030	3	19,044	NS
	*DQA1*05:05:01*	11	19,323	6	29,821	NS
	*DQA1*01:01:01*	8	20,269	4	22,623	NS
	*DQA1*01:02:01*	27	21,265	8	24,577	NS

	*DQA1*04:01:01*	10	34,300	7	28,479	NS
	*DQA1*01:03:01*	35	36,674	7	31,111	NS
	*DQA1*03:02:01*	27	52,211	6	54,126	NS
	*DQA1*03:01:01*	18	56,296	3	67,992	NS
	*DQA1*03:03:01*	25	57,501	7	52,527	NS
HLA-DQB1	*DQB1*04:02:01*	15	17,371	5	27,719	3.7×10^{-4}
	*DQB1*03:03:02*	31	20,990	9	19,194	NS
	*DQB1*03:02:01*	23	21,180	3	26,275	NS
	*DQB1*04:01:01*	29	22,424	9	23,654	NS
	*DQB1*03:01:01*	38	25,983	10	22,364	NS
	*DQB1*06:01:01*	42	40,730	12	43,273	NS
	*DQB1*05:01:01*	18	64,706	8	63,027	NS
	*DQB1*06:04:01*	11	72,682	7	86,514	NS
	*DQB1*05:03:01*	17	80,059	4	86,679	NS
	*DQB1*06:02:01*	18	83,443	10	84,605	NS
HLA-DRA	*DRA*01:01:01*	80	148,601	24	162,713	2.8×10^{-3}
	*DRA*01:02:02*	80	153,460	24	164,146	NS
HLA-DRB1	*DRB1*08:03:02*	22	99,464	4	107,726	NS
	*DRB1*08:02:01*	11	105,105	8	108,493	NS
	*DRB1*15:01:01*	18	108,733	10	102,839	NS
	*DRB1*04:05:01*	27	128,746	10	146,549	1.7×10^{-5}
	*DRB1*15:02:01*	24	131,182	8	115,590	NS
	*DRB1*13:02:01*	14	142,335	9	139,370	NS
	*DRB1*01:01:01*	12	143,227	8	123,199	8.0×10^{-4}
	*DRB1*12:01:01*	11	153,970	4	142,530	NS
	*DRB1*09:01:02*	40	157,239	10	155,997	NS
HLA-DRB3	*DRB3*02:02:01*	27	26,715	6	28,528	NS
	*DRB3*03:01:01*	17	36,398	8	41,339	NS
HLA-DRB4	*DRB4*01:03:01*	28	57,689	5	66,047	NS
	*DRB4*01:03:02*	13	82,362	3	87,839	NS
HLA-DRB5	*DRB5*01:01:01*	14	52,615	6	65,555	NS
	*DRB5*01:02*	21	57,684	6	64,935	NS

NS. indicates "not significant". Red letter indicates a low *p* -value under 0.001.
