# Supplementary material for: Capturing Differential Allele-Level Expression and Genotypes of All Classical HLA Loci and Haplotypes by a New Capture RNA-Seq Method
Source: Front Immunol. 2020 May 29;11:941. doi: 10.3389/fimmu.2020.00941 (PMC7272581; doi:10.3389/fimmu.2020.00941)
Supplement: Supplementary file 8 [file Data_Sheet_1.PDF]

Figure S1

A

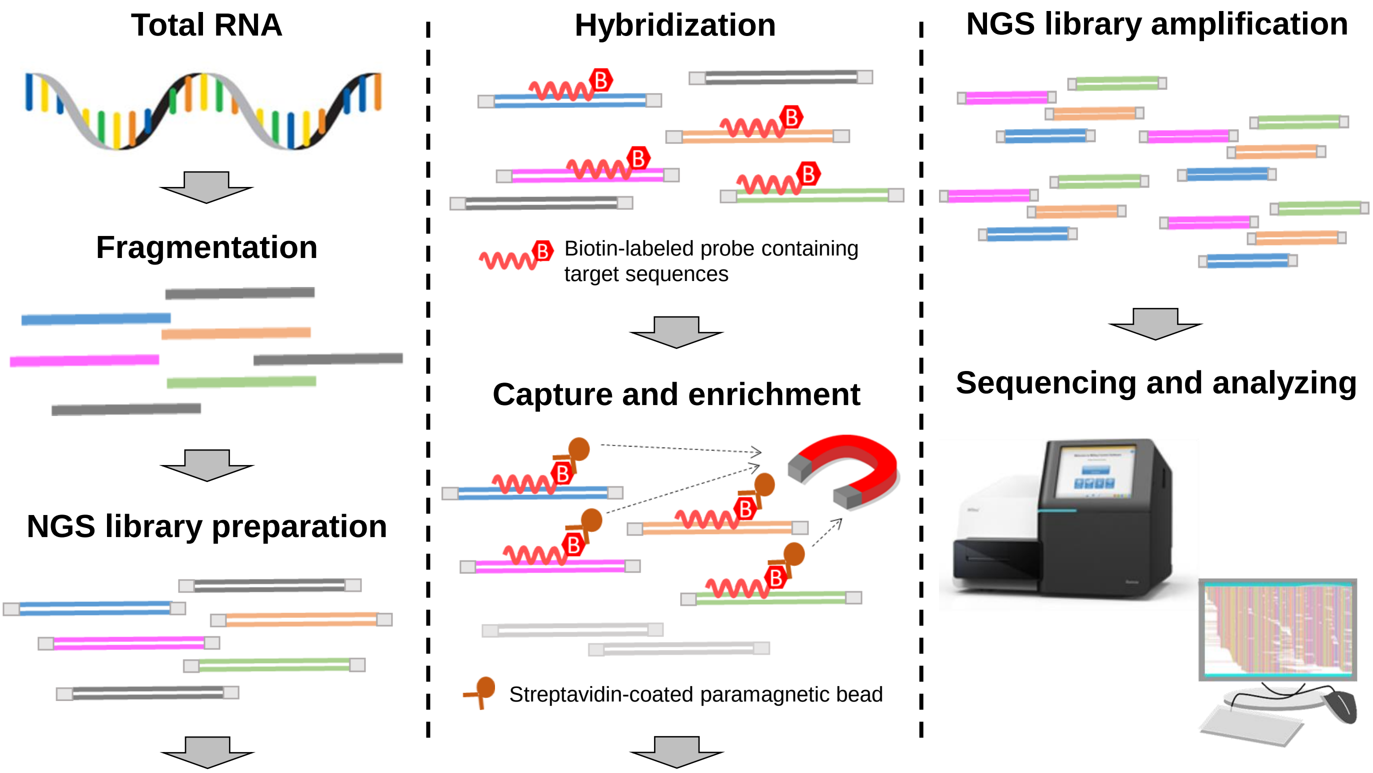

Total RNA
Fragmentation
NGS library preparation
Hybridization
Biotin-labeled probe containing target sequences
Capture and enrichment
Streptavidin-coated paramagnetic bead
NGS library amplification
Sequencing and analyzing

Figure S1

B

Mapping the reads for the HLA references using Reference Mapper

↓

Normalization of the read numbers

	HLA-A Ref. length: 546 bp		*HLA-B* Ref. length: 546 bp		*HLA-C* Ref. length: 546 bp	
Formula	Mapped reads	Mapped reads / kb	Mapped reads	Mapped reads / kb	Mapped reads	Mapped reads / kb
	/	(1) / 546 x 1,000=(3)<br>(2) / 546 x 1,000=(4)	/	(5) / 546 x 1,000=(6)<br>(7) / 546 x 1,000=(8)	/	(9) / 546 x 1,000=(10)<br>(11) / 546 x 1,000=(12)
Example Allele1*	24,842 (1)	45,498 (3)	47,060 (5)	86,190 (7)	25,213 (9)	46,178 (11)
Example Allele2*	25,881 (2)	47,401 (4)	48,074 (6)	88,048 (8)	24,080 (10)	44,103 (12)

↓

			Normalized *HLA-A*	Normalized *HLA-B*	Normalized *HLA-C*
Formula	Sum of the mapped reads / kb of all loci	One million reads / sum of the mapped reads / kb of all loci	One million reads / sum of the mapped reads / kb of all loci x Mapped reads / kb in each locus		
	(3)+(4)+(7)+(8)+(11)+(12) =(13)	1,000,000/ (13)=(14)	(3)x(14)=(15)<br>(4)x(14)=(16)	(7)x(14)=(17)<br>(8)x(14)=(18)	(11)x(14)=(19)<br>(12)x(14)=(20)
Example Allele1*	357,418 (13)	2.7978 (14)	127,294 (15)	241,142 (17)	129,197 (19)
Example Allele2*			132,619 (16)	246,341 (18)	123,391 (20)

Figure S1

C

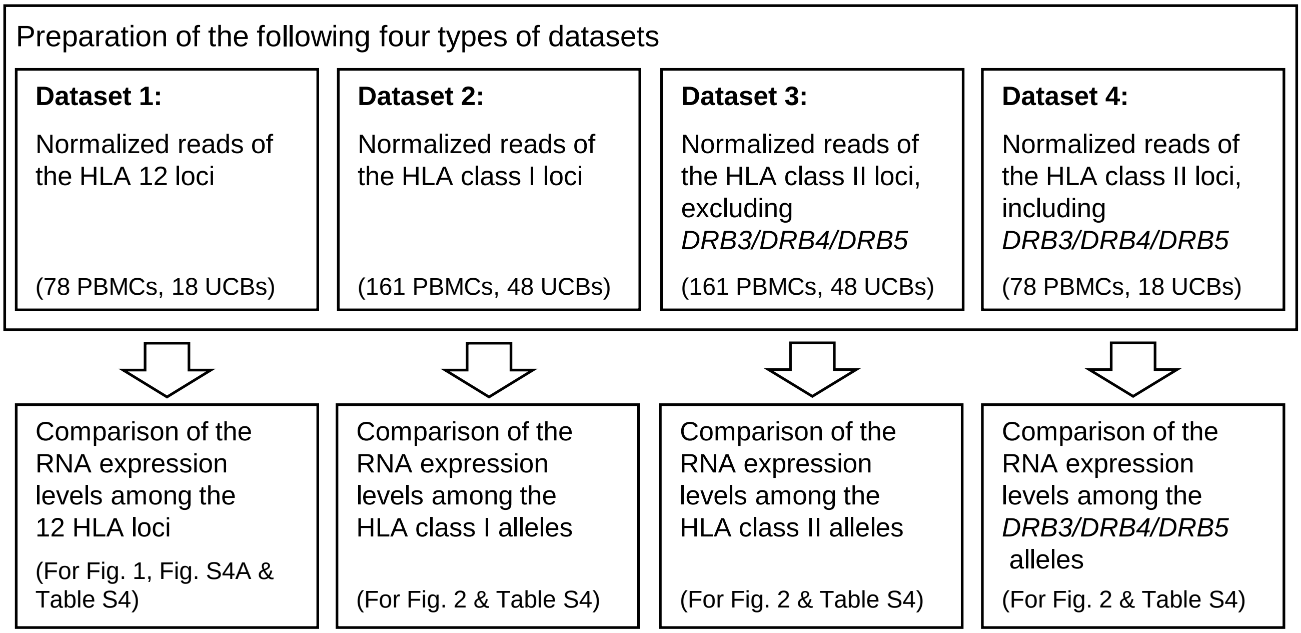

Figure S1. Workflow of the capture RNA-Seq method. (A), a schematic overview of NGS library construction from total RNA fragmentation and to output of sequence reads. (B), sequence read mapping to normalization of the reads with examples of mapping and normalizing *HLA-A*, *HLA-B* and *HLA-C* alleles and the number of sequencing reads. (C), preparation of four types of datasets for comparison of RNA expression levels among HLA loci and alleles. Classification for analyzed and excluded allele numbers based on the allelic information (Table S4 in Supplementary material).
