# Supplementary material for: Capturing Differential Allele-Level Expression and Genotypes of All Classical HLA Loci and Haplotypes by a New Capture RNA-Seq Method
Source: Front Immunol. 2020 May 29;11:941. doi: 10.3389/fimmu.2020.00941 (PMC7272581; doi:10.3389/fimmu.2020.00941)
Supplement: Supplementary file 9 [file Data_Sheet_2.PDF]

**Figure S2**

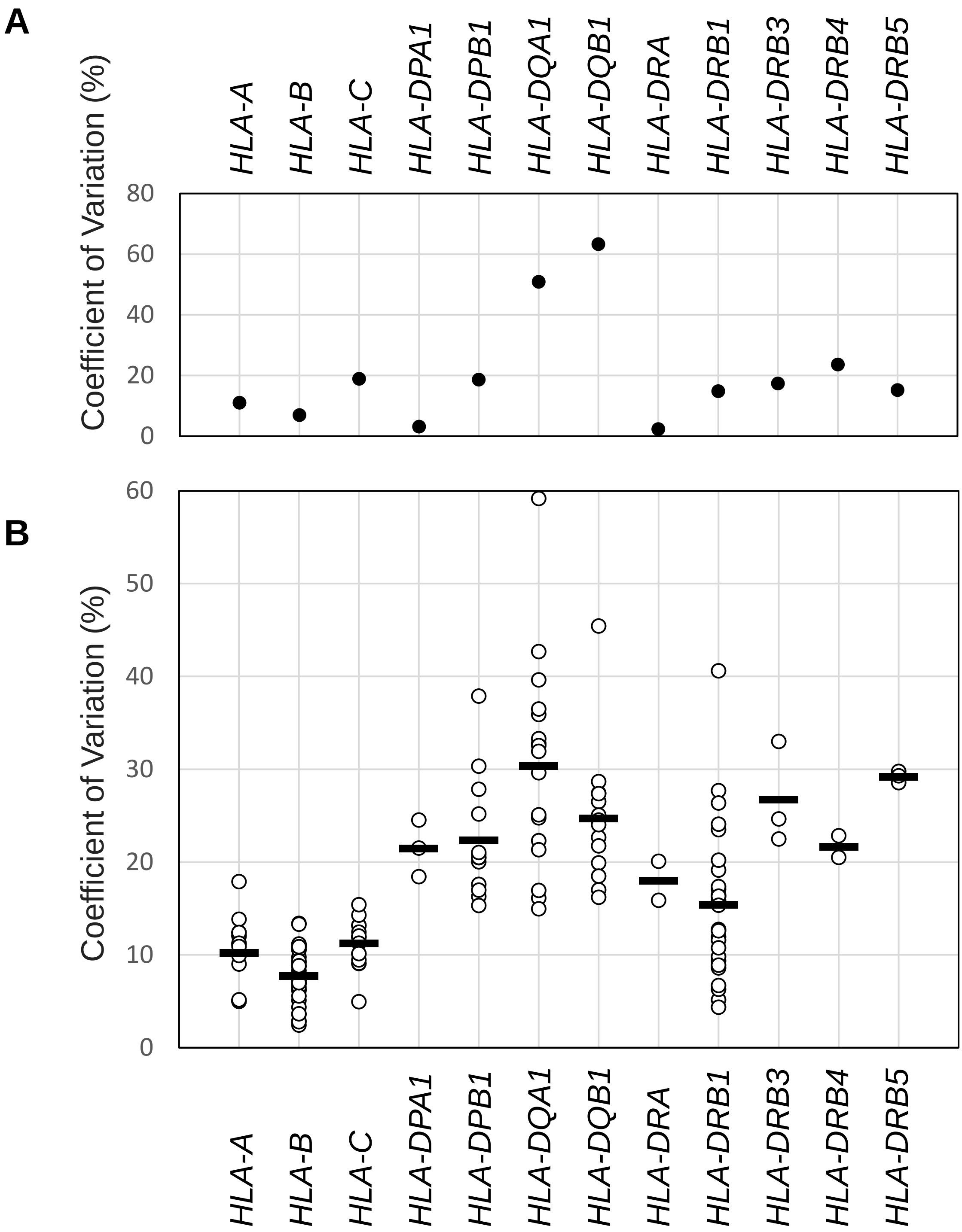


**Figure S2. Comparison of relative deviations in the levels of RNA expression among different alleles and different individuals.** (A) Comparison of read-number variations among different alleles at each locus. The sample standard deviation and average of medians of normalized read numbers of each alleles were calculated, and the coefficients of variation, plotted on the Y axis, were derived from the above standard deviations divided by the average medians at 12 loci. (B) Comparison of read-number variations among different individuals with the identical alleles. The coefficients of variation of read numbers of individual samples were calculated for individual alleles and plotted as open circles at each locus, with bars indicating means.
