# Supplementary material for: Capturing Differential Allele-Level Expression and Genotypes of All Classical HLA Loci and Haplotypes by a New Capture RNA-Seq Method
Source: Front Immunol. 2020 May 29;11:941. doi: 10.3389/fimmu.2020.00941 (PMC7272581; doi:10.3389/fimmu.2020.00941)
Supplement: Supplementary file 10 [file Data_Sheet_3.PDF]

Figure S3

A

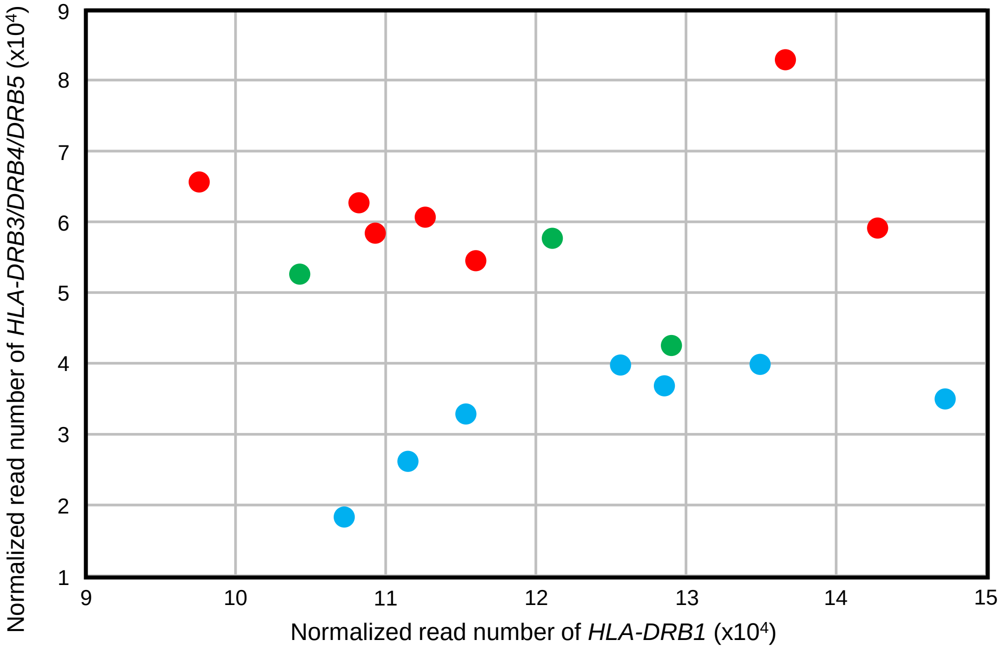

Normalized read number of *HLA-DRB3/DRB4/DRB5* (x10⁴)
9
8
7
6
5
4
3
2
1
9
10
11
12
13
14
15
Normalized read number of *HLA-DRB1* (x10⁴)

Figure S3

B

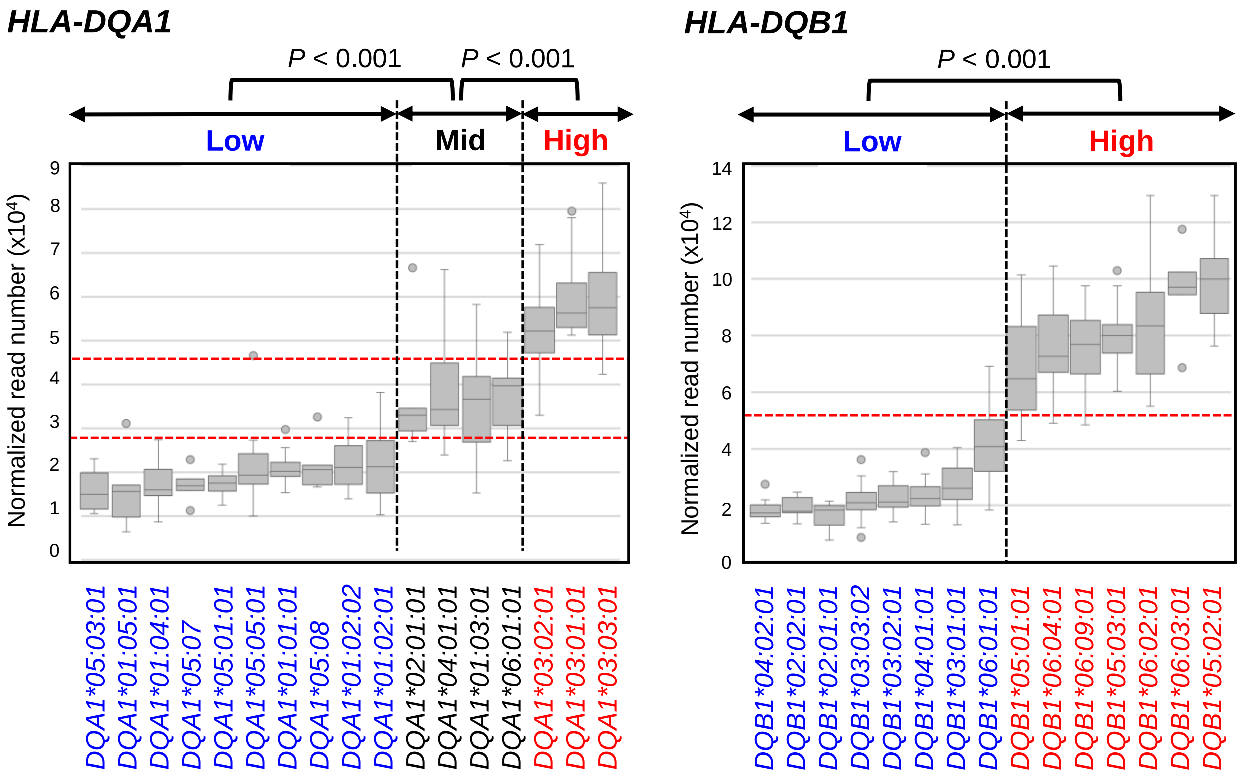

RNA expression level in *HLA-DQA1*	RNA expression level in *HLA-DQB1*	Frequency of DQ haplotypes in Japanese population	Frequency of DQ haplotypes in Worldwide populations
High	Low	40.5%	16.9%
Mid	Low	26.4%	17.3%
Low	Low	17.7%	25.2%
Low	High	13.6%	33.0%
Mid	High	1.8%	5.4%
High	High	0%	0%

Figure S3

C

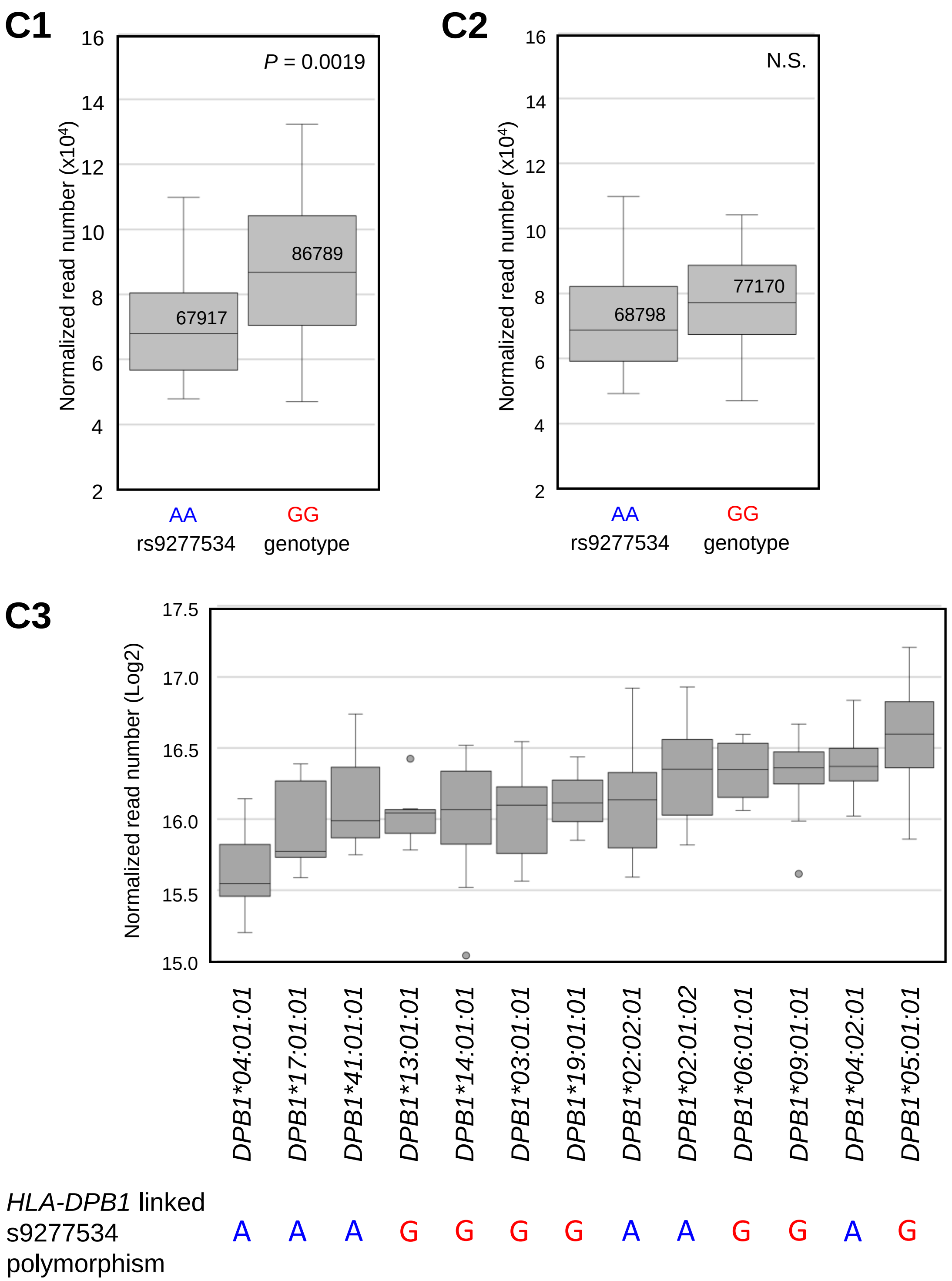

C1
P = 0.0019
Normalized read number (x10⁴)
16
14
12
10
8
6
4
2
67917
86789
AA
GG
rs9277534
genotype
C2
N.S.
Normalized read number (x10⁴)
68798
77170
AA
GG
rs9277534
genotype
C3
Normalized read number (Log2)
17.5
17.0
16.5
16.0
15.5
15.0
DPB1*04:01:01
DPB1*17:01:01
DPB1*41:01:01
DPB1*13:01:01
DPB1*14:01:01
DPB1*03:01:01
DPB1*19:01:01
DPB1*02:02:01
DPB1*02:01:02
DPB1*06:01:01
DPB1*09:01:01
DPB1*04:02:01
DPB1*05:01:01
HLA-DPB1 linked
s9277534
polymorphism
A A A G G G G A A G G A G

Figure S3

D

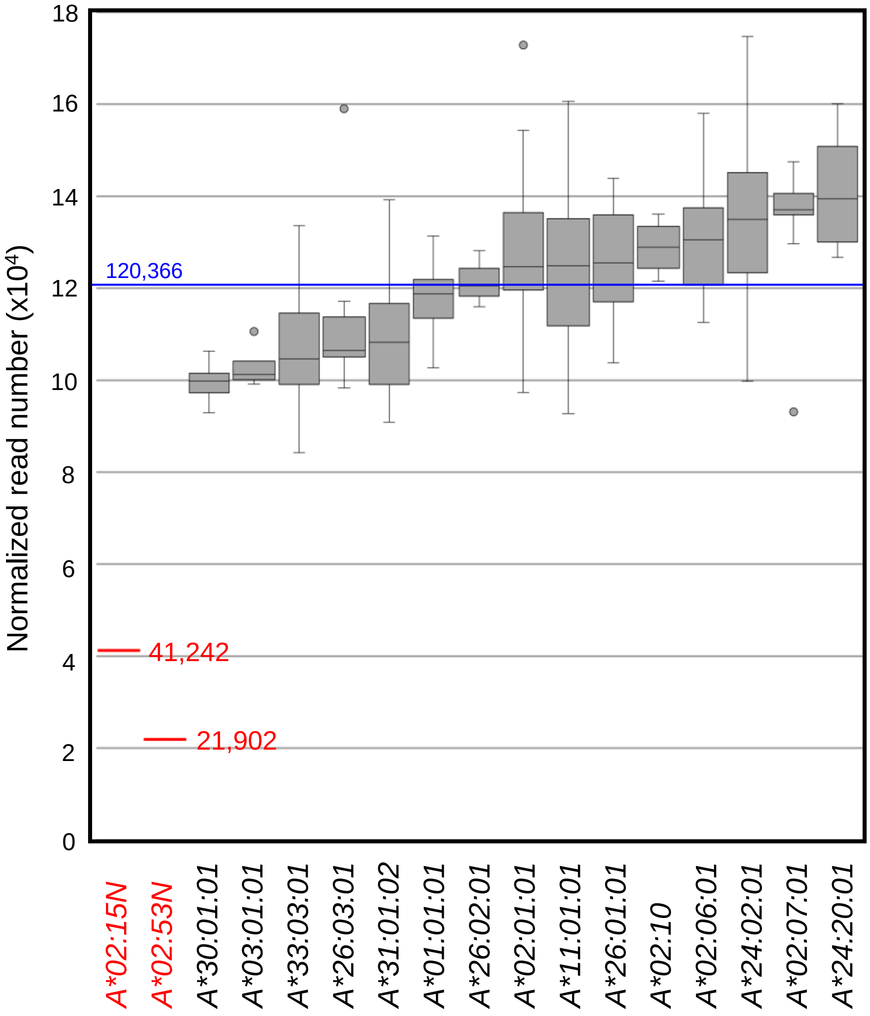
Normalized read number (x10⁴)
0
2
4
6
8
10
12
14
16
18
120,366
41,242
21,902
A*02:15N
A*02:53N
A*30:01:01
A*03:01:01
A*33:03:01
A*26:03:01
A*31:01:02
A*01:01:01
A*26:02:01
A*02:01:01
A*11:01:01
A*26:01:01
A*02:10
A*02:06:01
A*24:02:01
A*02:07:01
A*24:20:01

Figure S3

E

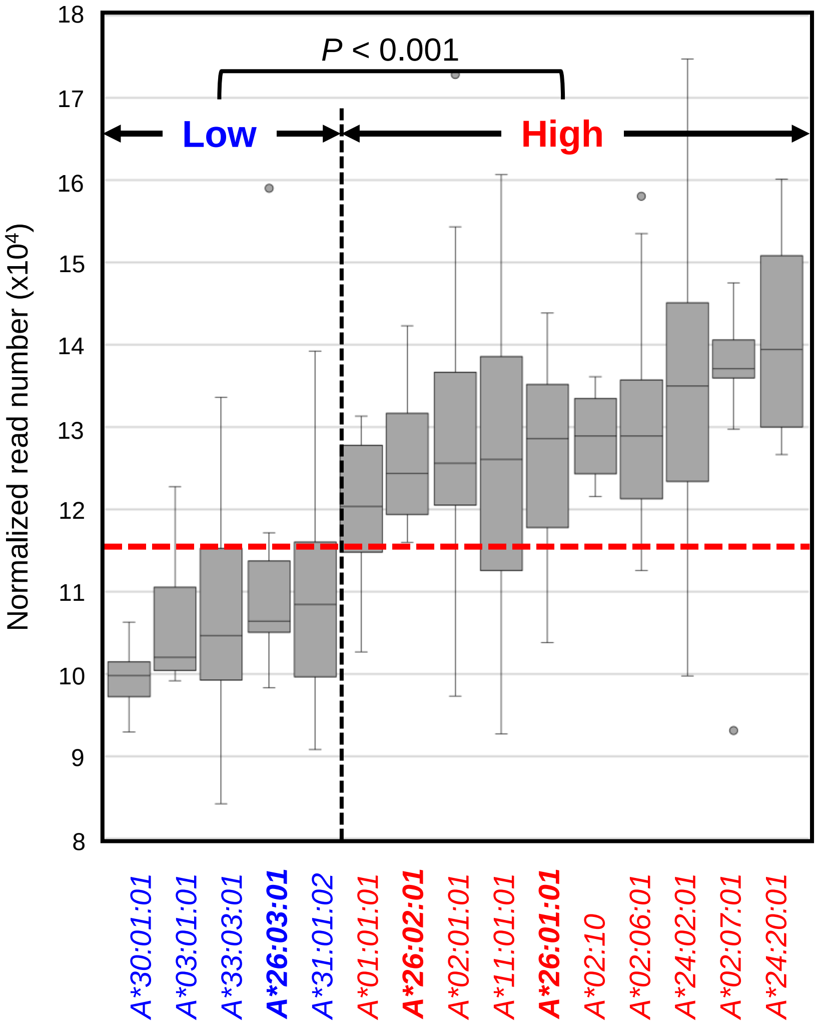

P < 0.001
Low
High
Normalized read number (x10^4)
18
17
16
15
14
13
12
11
10
9
8
A*30:01:01
A*03:01:01
A*33:03:01
A*26:03:01
A*31:01:02
A*01:01:01
A*26:02:01
A*02:01:01
A*11:01:01
A*26:01:01
A*02:10
A*02:06:01
A*24:02:01
A*02:07:01
A*24:20:01

Figure S3

Figure S3. Relationships between HLA gene and allele expression levels. (A) shows the comparative RNA levels expressed by *HLA-DRB1* (horizontal axis) and *HLA-DRB3*/*DRB4*/*DRB5* (vertical axis) of 17 class II haplotypes indicated in Table S5. Blue, red and green circles indicate expression levels of *DRB1-DRB3*, *DRB1-DRB4* and *DRB1-DRB5*, respectively. (B) shows the RNA expression levels (as normalized read numbers on the vertical axis) of *HLA-DQA1* (left) and *HLA-DQB1* (right) alleles (horizontal axis) in PBMC samples with the expression levels graded as low (blue), intermediate or mid (black) and high (red). The statistical significances ($P < 0.001$) between the grades of low, mid or high are indicated by double arrows and the barred horizontal lines. Horizontal lines within the boxes indicate the median in each expressed allele. The HLA-DQ haplotype frequency of Japanese population in the bottom table is sourced from the HLA laboratory HLA allele frequency data (http://hla.or.jp/med/frequency_search/en/haplo/) and the 17th IHIW NGS HLA genotyping Data (http://17ihiw.org/17th-ihiw-ngs-hla-data/). (C) shows the differential RNA expression levels of *HLA-DPB1* alleles and genotypes of rs9277534. (C1) and (C2): RNA expression levels (as normalized read numbers on the vertical axis) for AA (blue) and GG (red) genotypes of rs9277534 and for AA and GG genotypes, excluding outlier alleles *DPB1*04:01:01* and *DPB1*05:01:01*, of rs9277534 (horizontal axis). Value in the box of the box-and-whisker diagram indicates average median of the normalized read numbers. (C3): Linkage of the *HLA-DPB1* and A (blue) and G (red) alleles of rs9277534 locus. (D) shows the RNA expression levels (as normalized read numbers on the vertical axis) of *HLA-A* (left) and *HLA-C* (right) alleles (horizontal axis) in PBMC samples with an emphasis on the expression of null or low expressed HLA alleles (red). Read numbers of *A*02:15N* and *A*02:53N* were plotted on the *HLA-A* plot as shown in Figure 2. The average median of the loci and read numbers of the null and low expressed HLA alleles are indicated by blue and red letters. Horizontal lines in the boxes indicate the median for each expressed allele. (E) shows the RNA expression levels (as normalized read numbers on the vertical axis) of *HLA-A* alleles (horizontal axis) in PBMC graded as low (blue) or high (red). The statistical significance between the low- and high-grade expressions is indicated by double arrows and the barred horizontal line beneath ($P < 0.001$). *A*26:01:01*, *A*26:02:01* and *A*26:03:01* are indicated by bold letters. Horizontal lines in the boxes indicate the median in each expressed allele.
