# Supplementary material for: Capturing Differential Allele-Level Expression and Genotypes of All Classical HLA Loci and Haplotypes by a New Capture RNA-Seq Method
Source: Front Immunol. 2020 May 29;11:941. doi: 10.3389/fimmu.2020.00941 (PMC7272581; doi:10.3389/fimmu.2020.00941)
Supplement: Supplementary file 11 [file Data_Sheet_4.PDF]

Figure S4

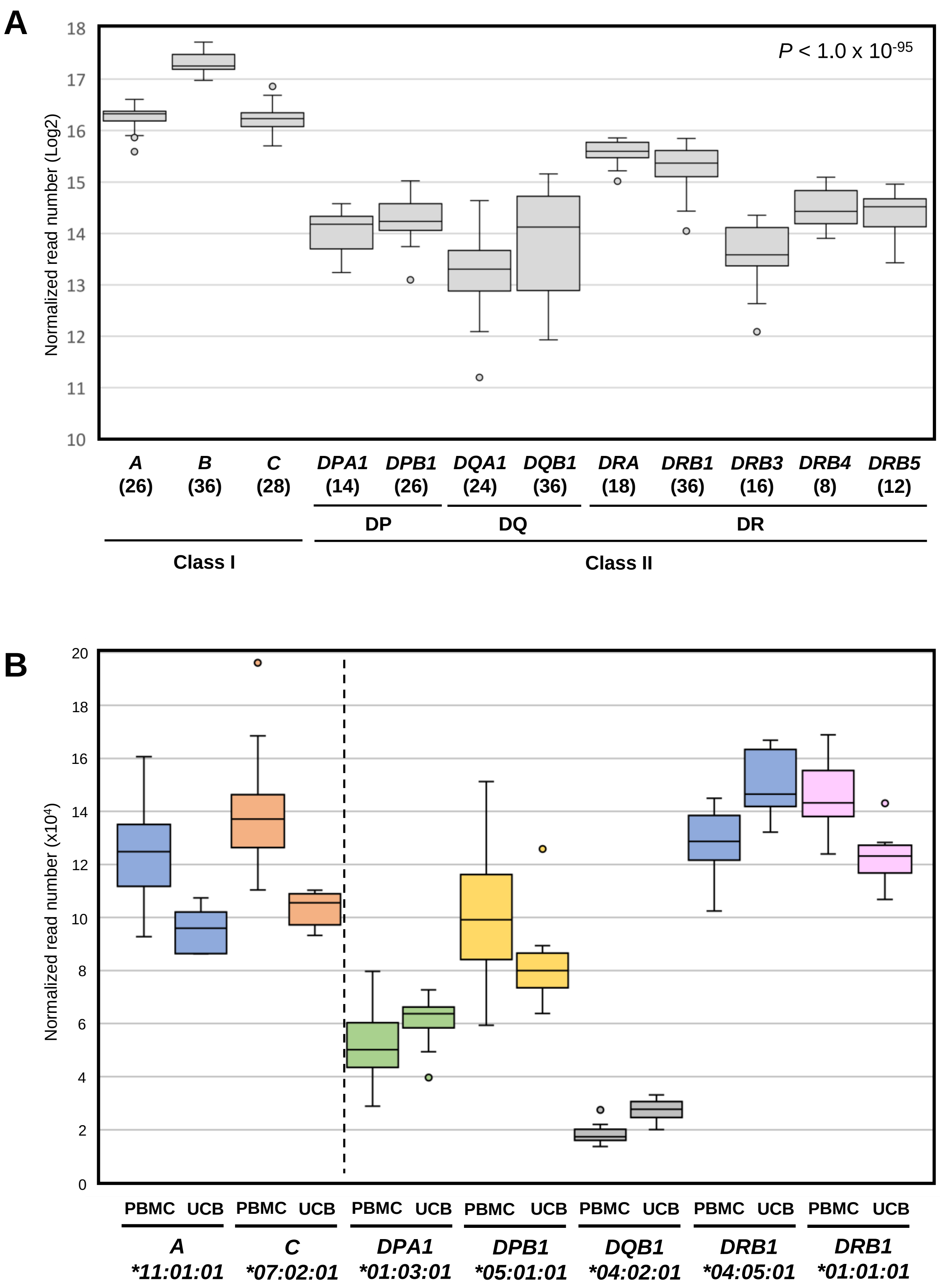
A
B
P < 1.0 x 10-95
Normalized read number (Log2)
18
17
16
15
14
13
12
11
10
A (26)
B (36)
C (28)
DPA1 (14)
DPB1 (26)
DQA1 (24)
DQB1 (36)
DRA (18)
DRB1 (36)
DRB3 (16)
DRB4 (8)
DRB5 (12)
DP
DQ
DR
Class I
Class II
Normalized read number (x10⁴)
20
18
16
14
12
10
8
6
4
2
0
PBMC UCB
A *11:01:01
C *07:02:01
DPA1 *01:03:01
DPB1 *05:01:01
DQB1 *04:02:01
DRB1 *04:05:01
DRB1 *01:01:01

Figure S4

Figure S4. RNA levels expressed by 12 HLA loci in UCB (A), and a comparison of the largest quantitative RNA differences expressed by HLA alleles of PBMC and UCB (B). (A) shows RNA expression levels of 12 HLA loci in UCB measured by capture RNA-Seq. This box-and-whisker diagram using the results of dataset 1 from the sequence reads of 18 UCB samples (Figure S1C). Vertical axis indicates normalized read numbers (x10^4) calculated by the method described in Figure S1B. Horizontal axis indicates the 12 classical class I and class II HLA loci. Circles within the matrix indicate normalized read numbers (log2) of each sample, and horizontal lines in the box indicate the expressed median of 18 samples. Parenthesis below the locus name indicates the number of individual points plotted per each locus. (B) shows the statistically significant differences (P <0.001) between PBMC and UCB samples as a box-and-whisker diagram for the largest RNA levels expressed by shared alleles of each HLA locus. Vertical axis indicates normalized read numbers (x10^4) and horizontal axis shows the seven significantly different class I and class II HLA alleles and loci. Horizontal lines in the boxes of the matrix indicate expressed median of the PBMC and UCB samples. Horizontal axis shows the seven significantly different class I and class II HLA alleles and loci.
