# Supplementary material for: Capturing Differential Allele-Level Expression and Genotypes of All Classical HLA Loci and Haplotypes by a New Capture RNA-Seq Method
Source: Front Immunol. 2020 May 29;11:941. doi: 10.3389/fimmu.2020.00941 (PMC7272581; doi:10.3389/fimmu.2020.00941)
Supplement: Supplementary file 12 [file Data_Sheet_5.PDF]

Figure S5

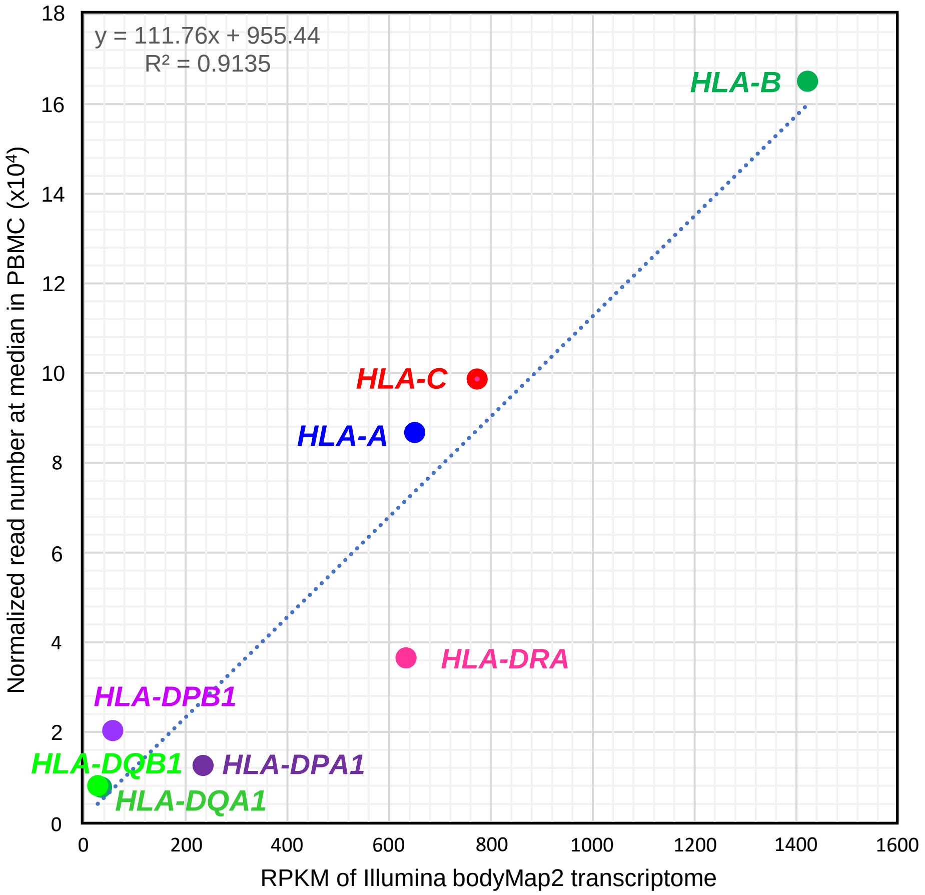

Figure S5. Comparison between the class I and class II HLA gene expression results using RNA capture probes and Illumina RNA-seq for PBMC samples in our study and those of the Illumina RNA-seq data for the bodyMap2 transcriptome. Normalized read numbers (10^4) at median for PBMC samples and RPKM values at median of white blood cell samples of the Illumina bodyMap2 transcriptome were plotted in the vertical and horizontal axis, respectively.
