# Supplementary material for: Capturing Differential Allele-Level Expression and Genotypes of All Classical HLA Loci and Haplotypes by a New Capture RNA-Seq Method
Source: Front Immunol. 2020 May 29;11:941. doi: 10.3389/fimmu.2020.00941 (PMC7272581; doi:10.3389/fimmu.2020.00941)
Supplement: Supplementary file 13 [file Data_Sheet_6.PDF]

Figure S6

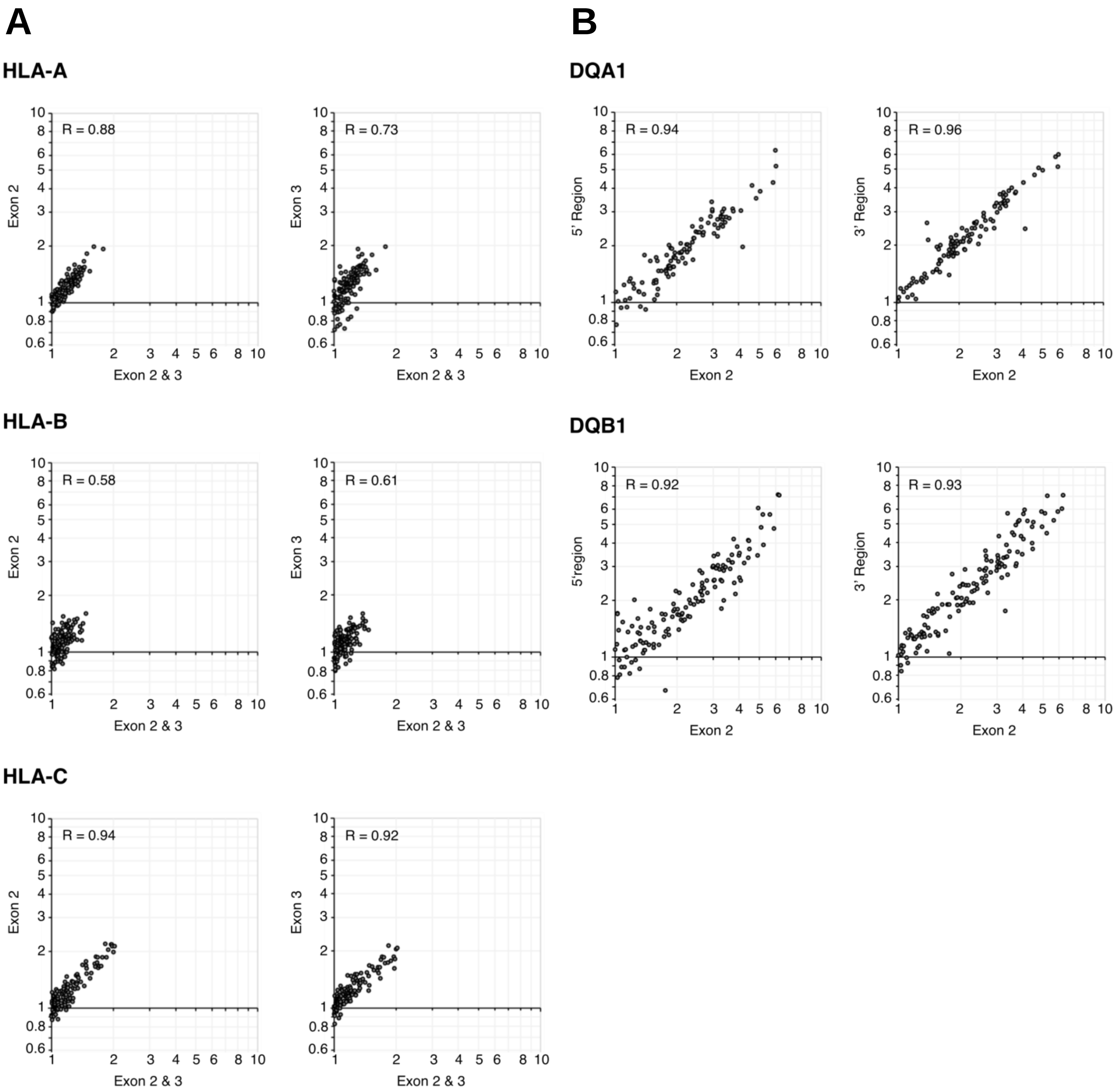

Figure S6. Allelic ratios of read numbers of individual samples at the HLA class I and II loci. Allelic ratios of the normalized read numbers described in this paper at the class I and class II loci were calculated for individual samples, and the calculated ratios were plotted on the X axes of the graphs of the HLA-A, B and C genes **(A)** and the DQAI and DQBI genes **(B)**. The allelic read-number ratios of non-overlapping sub-regions of the original quantitation targets were also calculated for individual samples, and the ratios were plotted on the Y axes of the graphs. We then evaluated the correlation between the allelic ratios of the original targets and those of the sub-regions, and the correlation coefficient values were calculated as shown on each graph panels.
